# Novel self-assembling conjugates as vectors for agrochemical delivery

**DOI:** 10.1186/s12951-018-0423-5

**Published:** 2018-11-21

**Authors:** Pavani P. Nadiminti, Qingtao Liu, Lavanya K. Vanjari, Yao D. Dong, Ben J. Boyd, David M. Cahill

**Affiliations:** 1 School of Life and Environmental Sciences, Deakin University, Waurn Ponds Campus, Geelong, VIC 3217 Australia; 2 Drug Delivery, Disposition and Dynamics, Monash Institute of Pharmaceutical Sciences, Monash University, Parkville Campus, 381 Royal Parade, Parkville, VIC 3052 Australia; 3 ARC Centre of Excellence in Convergent Bio-Nano Science and Technology, Monash Institute of Pharmaceutical Sciences, Monash University, Parkville Campus, 381 Royal Parade, Parkville, VIC 3052 Australia

**Keywords:** Self-assembly, Picloram, Herbicides, *Arabidopsis thaliana*, Surfactants, Weeds

## Abstract

**Background:**

Modern agricultural practises rely on surfactant-based spray applications to eliminate weeds in crops. The wide spread and indiscriminate use of surfactants may result in a number of deleterious effects that are not limited to impacts on the crop and surrounding farm eco-system but include effects on human health. To provide a safer alternative to the use of surfactant-based formulations, we have synthesised a novel, self-assembling herbicide conjugate for the delivery of a broad leaf herbicide, picloram.

**Results:**

The synthesized self-assembling amphiphile–picloram (SAP) conjugate has three extending arms: a lipophilic lauryl chain, a hydrophilic polyethylene glycol chain and the amphiphobic agrochemical active picloram. We propose that the SAP conjugate maintains its colloidal stability by quickly transitioning between micellar and inverse micellar phases in hydrophilic and lipophilic environments respectively. The SAP conjugate provides the advantage of a phase structure that enables enhanced interaction with the hydrophobic epicuticular wax surface of the leaf. We have investigated the herbicidal efficiency of the SAP conjugate compared against that of commercial picloram formulations using the model plant *Arabidopsis thaliana* and found that when tested at agriculturally relevant doses between 0.58 and 11.70 mM a dose-dependent herbicidal effect with comparable kill rates was evident.

**Conclusion:**

Though self-assembling drug carriers are not new to the pharmaceutical industry their use for the delivery of agrochemicals shows great promise but is largely unexplored. We have shown that SAP may be used as an alternative to current surfactant-based agrochemical formulations and has the potential to shift present practises towards a more sustainable approach.

**Electronic supplementary material:**

The online version of this article (10.1186/s12951-018-0423-5) contains supplementary material, which is available to authorized users.

## Background

Common agricultural practises aimed at improving plant health and crop yield are heavily dependent on the use of surfactant-based agrochemical formulations. Such formulations are known for their off-target toxicity [1], lead to the over-use of chemicals [2] and may result in crop phytotoxicity [3, 4] ultimately resulting in yield loses. In addition, traditional agrochemicals may significantly contribute to pollution of the environment and can be deleterious to human health [5, 6]. To overcome these unintended impacts new strategies for agrochemical delivery are under constant exploration [7, 8]. One such innovative strategy is the use of nanotechnology, which has opened new vistas for the delivery of agrochemicals to plants [9]. Unlike traditional agrochemicals, research with the model plant species, *Arabidopsis thaliana,* showed that mesoporous silica nanoparticles (MSNs), can deliver the phytohormone and disease resistance inducer, salicylic acid, *in*-*planta* only when redox stress is high [10]. Also, MSNs were shown to be able to deliver the herbicide 2,4-dichlorophenoxyacetic acid (2,4-D) to control the growth of model weeds while simultaneously reducing soil contamination [11]. In our recent work, lyotropic liquid crystalline (LC) phases were shown in laboratory and field studies to minimise crop phytotoxicity while efficiently delivering 2,4-D to eliminate weeds [12].

LC systems have been shown to have high pharmaceutical value for their ability to deliver a range of molecules such as curcumin [13], chlorhexidine [14] and insulin [15]. LC systems encompass a number of phases such as lamellar phases, hexagonal phases and inverse bicontinuous cubic phases. The latter phases can simply be formed by dispersion in water of polar lipids such as phytantriol, glyceryl monooleate and glyceryl monoelaidin in the presence of a stabiliser [16]. Such LC systems have recently been of interest also for the delivery of agrochemical actives to plants. LC systems in inverse bicontinuous cubic phase structure can interact with hydrophobic plant surfaces, prevent crop phytotoxicity and reduce off target toxicity effects [12, 17]. In field trials the practical use of a surfactant free LC system for the delivery of 2,4-D to kill wild radish (*Raphanus raphanistrum*) in a wheat (*Triticum aestivum*) crop was demonstrated [17]. However, their success was dependent on the amphiphobic agrochemical in use that often hampers the stability of the formulation [18–20].

Agrochemical industries employ several strategies to improve the stability of oil in water emulsions, for example, by encapsulation of the active ingredient [21], alkoxylation of the hydrophobic active ingredient [22] and the use of solid dispersing agents to stabilise the emulsion containing lipophilic actives [23]. In a recent report we demonstrated the use of an agrochemical conjugate for the formation of a stable self-assembling LC carrier system [8]. The linear-picloram conjugate was prepared by covalently bonding agrochemical actives such as picloram or 2,4-D to a lipid, which, when exposed to water in the presence of a pluronic stabiliser, formed a self-assembling LC system. The use of such formulation is restricted by the presence of amphiphobic actives that limit their encapsulation into the LC system and reduces the colloidal stability of the formulation [8]. The disadvantages of such systems presents us with the need for the development of an ecologically safe, self-assembling agrochemical formulation that can not only form a stable carrier emulsion but also can efficiently deliver the active to target plants.

The use of self-assembling liquid crystalline materials for the delivery of pharmaceutical actives is well described but has never been explored for the delivery of agrochemical actives [24]. We therefore, synthesized a novel self-assembling amphiphile–picloram (SAP) conjugate with three extending arms consisting of a lipophilic lauryl chain, hydrophilic polyethylene glycol (PEG) chain and an amphiphobic agrochemical active, (picloram in this case). Due to the presence of the extending arms, the SAP conjugate formed a colloidally stable formulation in the absence of surfactants, which enabled the conjugate to better interact with hydrophobic plant surfaces. To test the herbicidal efficacy of the formulation, the model plant species, *A. thaliana,* was treated with the SAP conjugate at agriculturally relevant doses. The herbicidal effect of the SAP formulation was found to be equal to that of surfactant-based picloram formulations and therefore has considerable potential as an alternative delivery system for agrochemicals.

## Methods

### Materials

Picloram (4-amino-3,5,6-trichloro-2-pyridinecarboxylic acid) and picloram-oleyl (PO) were the gift of Nufarm Ltd. (Laverton, Vic, Australia), *O*-(6-chlorobenzotriazol-1-yl)-*N,N,N′,N′*-tetramethyluronium hexafluorophosphate (HCTU), triisopropylamine, methoxypolyethylene glycol 350 (Me-EO_7_–OH), 1-bromododecane, 18-crown-6, sodium hydroxide and potassium carbonate anhydrous were purchased from Sigma-Aldrich (St. Louis, MO, USA). p-Toluenesulfonyl chloride (Ots-Cl) and 3,5-dihydroxybenzyl alcohol were purchased from TCL Co. Ltd. (Chuo-ku, Tokyo, Japan). Diethyl ether, chloroform, methanol, acetonitrile and magnesium sulphate anhydrous were purchased from Merck Pty. Ltd. (Kilsyth, Vic, Australia).

### Preparation of 3-(dodecyloxy)-5-(hydroxymethyl)phenol (2)

**Fig. 1:**
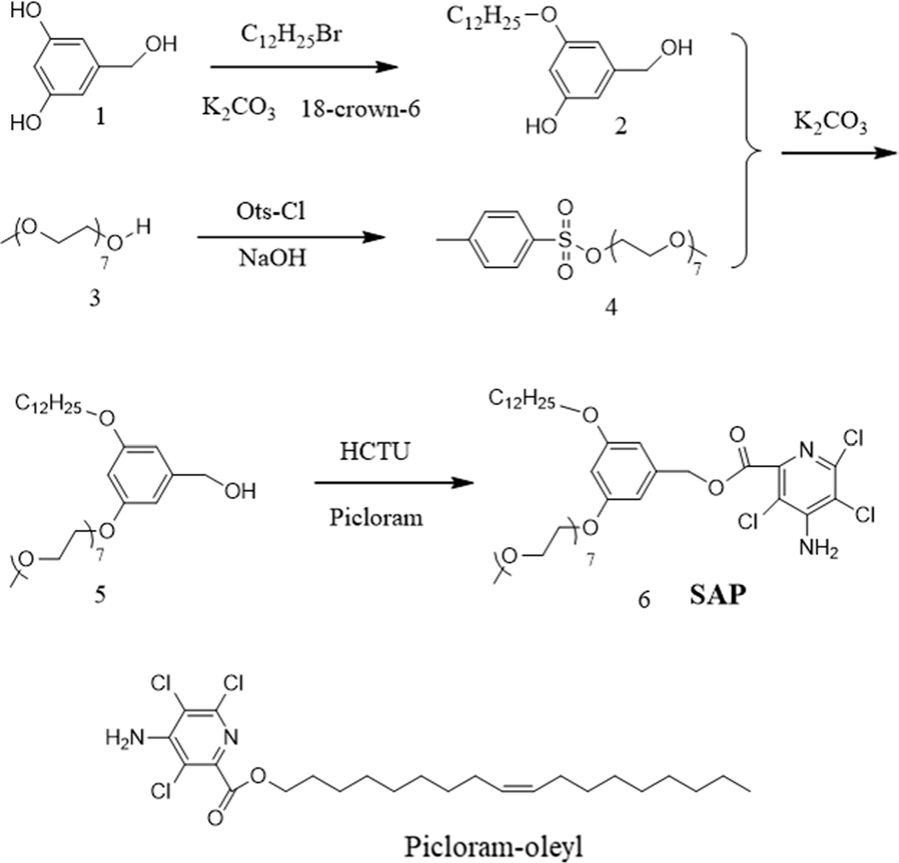
Chemical structure and synthesis route of the SAP conjugate

1.68 g of 3,5-dihydroxybenzyl alcohol (1) (12 mM), 2.49 g of 1-bromododecane (10 mM), 0.1 g of 18-crown-6 (1,4,7,10,13,16-hexaoxacyclooctadecane) and 4.14 g of K_2_CO_3_ (30 mM) were dissolved in 150 mL dimethylformamide (DMF) under continuous stirring in a 250 mL round-bottom flask. The mixture was refluxed for 8 h at 120 °C after transferring the round-bottom flask to an oil bath. The product was then dried using reduced pressure distillation and re-dissolved in chloroform to remove insoluble inorganic salts through filtration. The product was dried with a rotary vacuum evaporator and purified using column chromatography using a mixture of chloroform (90% v/v) and methanol (10% v/v) as the eluent. The final product was a white powder (yield was 67%) (Fig. 1) (Additional file 1).

### Preparation of Ots-EO_7_-Me (4)

20 mM Me-EO_7_–OH (3) solution in 8 mL tetrahydrofuran (THF) and 24 mmol NaOH aqueous solution (10% w/w) were mixed under continuous stirring in a 100 mL round-bottom flask and placed in an ice bath. While constantly, stirring 8 mL THF solution and 22 mM p-toluenesulfonyl chloride was slowly added to the round-bottom flask and stirred for another 2 h at room temperature. After transferring the reaction to 100 mL milli-Q water, the mixture was extracted thrice with 40 mL chloroform and was dried with 5 g anhydrous magnesium sulfate. The product was then filtered, to remove magnesium sulfate and dried to obtain a crude product. The crude product was further purified via column chromatography using a mixture of chloroform (90% v/v) and methanol (10% v/v) as the eluent. The purified product was a colourless oil (yield was 97%) (Fig. 1) (Additional file 2).

### Preparation of 3-(dodecyloxy)-5-(EO_7_)phenol (5)

0.2 mM Ots-EO_7_-Me (4), 0.24 mM 3-(dodecyloxy)-5-(hydroxymethyl)phenol (2) and 0.98 g of K_2_CO_3_ (0.7 mM) were dissolved in 100 mL DMF in a 250 mL round-bottom flask equipped with a magnetic bar and refluxed at 120 °C for eight hours. To obtain a crude product, the reaction mixture was filtered, to remove insoluble inorganic salts and was dried by reduced pressure distillation. The crude product was further purified by passing it through a chromatography column and was eluted with chloroform (95% v/v) and methanol (5% v/v) to obtain a pure product that was a dark yellow oil (yield is 57%) (Fig. 1) (Additional file 3).

### Preparation of 3-(dodecyloxy)-5-(EO_7_)benzyl-PIC (6)

0.48 g picloram (2 mM) was suspended in 70 mL acetonitrile in a 250 mL round-bottom flask equipped with a magnetic bar, then 1 g HCTU powder (2.4 mM) was added to the picloram suspension and stirred for 10 min. To this and at 10 min intervals, 1 mM of component-5 in 10 mL acetonitrile and 7.2 mM triisopropylamine (1.4 mL) were added with stirring overnight. The reaction solution was then dried to remove acetonitrile by rotary vacuum evaporation. The dried reaction mixture was extracted thrice with 40 mL diethyl ether. The combined diethyl ether extract was further dried to obtain a crude product that was passed through a chromatography column and then eluted using chloroform. The desired final product SAP conjugate, was a yellow oil (yield is 52%) (Fig. 1) (Additional files 4 and 5).

### The particle size of self-assembling amphiphile–picloram (SAP) conjugate in water

Particle size of the self-assembling amphiphile–picloram (SAP) conjugate was analysed at different concentrations in water using a zetasizer (Nano-ZS, Malvern Instruments, Malvern, UK) at 25 °C against the viscosity of pure water. Automated settings were used to take size measurements in low-volume cuvettes.

### The critical micelle concentration of SAP conjugate in water

The critical micelle concentration (CMC) of SAP conjugate in water was measured using a fluorescent probe method [25]. For this, aqueous solutions of pyrene and SAP conjugate were prepared where the concentration of pyrene was kept constant at 2 µM while that of the SAP conjugate ranged between 0 and 1.2 mM. The fluorescence emission intensity of pyrene in the samples at 370 nm (*I*_*1*_) and 380 nm (*I*_*3*_), was recorded using a Perkin Elmer Enspire^®^ (PerkinElmer Australia, Vic, Australia) multimode plate reader (excitation wavelength was at 330 nm). To obtain the CMC, the ratio of fluorescence emission intensity at *I*_*1*_ and *I*_*3*_ was plotted against the concentration of SAP conjugate, where the inflection point represents CMC.

### The water and oil contact angle of SAP conjugate cast membrane

The water and oil contact angle (CA) of a cast film of the SAP conjugate was measured using a goniometer (KSV CAM 101, Biolin Scientific AB, Västra Frölunda, Sweden).

### The morphology of SAP conjugate in water

To investigate the internal nanostructure of the SAP conjugate in water transmission electron microscopy (TEM) was performed. For this, a dispersion of SAP conjugate prepared in water was sonicated for 30 min and stored overnight. A ten-µl drop of the SAP conjugate aqueous solution was placed on a copper grid (ProSciTech, Qld, Australia), and allowed to dry at room temperature. TEM images were then collected of the grid using a JEOL 2100 TEM (JOEL Ltd., Tokyo, Japan) operating at 200 kV.

### Plant growth and maintenance

To test the herbicidal effect of various formulations, 3-week-old, *A. thaliana* ecotype Col-0 plants were used. Seeds were surface sterilised as previously described and were transferred to 90 mm Petri plates containing Murashige and Skoog basal medium (Sigma-Aldrich, Sydney, NSW, Australia) containing 30% w/v sucrose, 0.8% w/v bacteriological agar and adjusted to pH 5.7 [26]. The seeds within the Petri plates were stratified at 4 °C for 48 h and the plates then moved to a growth cabinet (Thermoline Scientific, Wetherill Park, NSW, Australia) for 14 days, under the conditions previously described [26].

### Determination of herbicidal effect of picloram

To mimic the herbicidal constituents that are routinely used in agricultural situations, picloram, empimin (an anionic sodium di(2-ethylhexyl) sulfosuccinate), empigen (a cationic and amphoteric betaine C12 − C14 alkyl dimethyl) (all gifts of Nufarm Australia Limited, Vic, Australia) and pluronic F127 (a nonionic polyoxyethylene–polyoxypropylene block co-polymer, Sigma-Aldrich, Australia) were diluted in water to obtain different formulations. A series of range finding experiments were carried out prior to the collection of data from duplicated experiments for all of the following treatment combinations. The formulations tested contained picloram at agriculturally relevant concentrations in the range 4.14 × 10^−5^ mM [1.0 × 10^−6^ % (w/v)] − 4.14 × 10^−1^ mM [1.0 × 10^−2^ % (w/v)] and mixed with one of the commercial surfactants empimin, empigen or pluronic F127 at 0.01% v/v [12]. To obtain a uniform coverage of leaves the formulations were individually spray-applied using a hand held atomiser (New directions, Sydney, NSW, Australia) to the adaxial surface of leaves of three *A. thaliana* plants per treatment. The plants were treated on a bench within the laboratory at room temperature and then left to dry (approximately 2 h) and were then transferred to a growth cabinet for observations and measurement of any herbicidal effect. Each plant was imaged three and seven days after spray application and assessed against control plants treated either with distilled water or with the corresponding surfactant formulation (n = 3 for each replicate) [12]. Commercial statistical software (IBM SPSS Version 19; IBM Australia Ltd., St Leonards NSW, Australia) was used to perform Duncan’s posthoc test on the normally distributed data.

### Herbicidal effect of the SAP conjugate

To test the efficiency of the SAP conjugate, it was diluted to agriculturally relevant doses in water to final concentrations of 0.585 mM [0.05% (w/v)], 1.17 mM [0.1% (w/v)], 2.34 mM [0.2% (w/v)], 5.85 mM [0.5% (w/v)] and 11.70 mM [1.0% (w/v)] and spray applied to *A. thaliana* as described above. Water treated controls were set up; picloram and picloram oleyl (PO) were simultaneously made in water to match the concentrations of the conjugate and were similarly sprayed on to *A. thaliana* plants [12, 17]. Blind assessment is a widely accepted and robust procedure for the assessment of phytotoxic effect induced by herbicidal spray applications [27]. For our work, three independent blind assessors separately ranked all the photographs collected for plants after day three and seven spray treatments on a scale of zero to five (n = 3 for each replicate). As the ranked data was uniformly distributed, it was subjected to Duncan’s posthoc testing (IBM SPSS Version 19, NSW, Australia) and the experiments were performed in triplicate.

## Results

### The colloidal property of the SAP conjugate in water

**Fig. 2:**
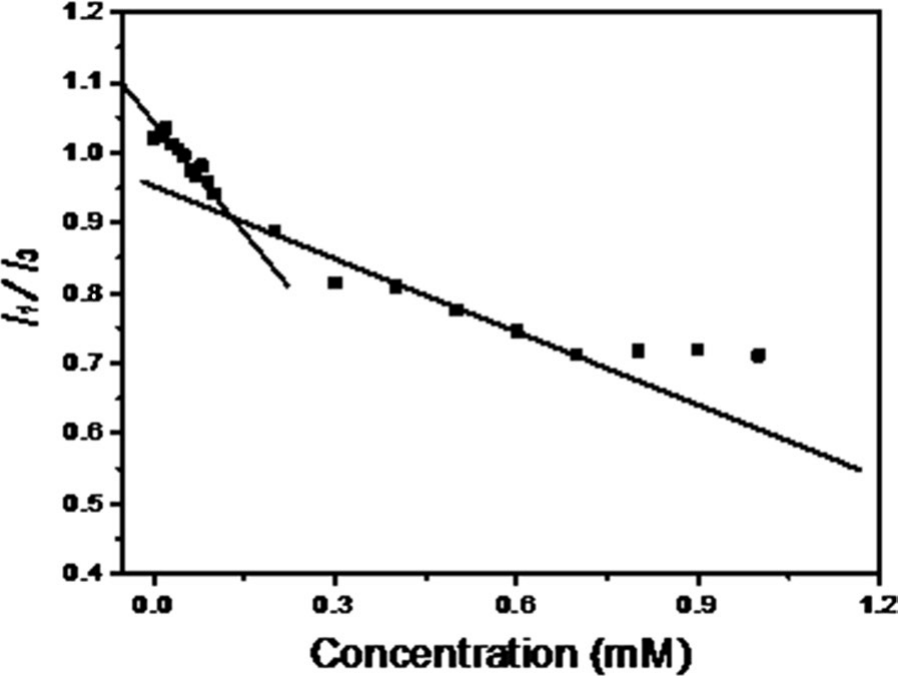
Fluorescence intensity ratios of first and third emission peaks of pyrene versus SAP conjugate concentration. The concentration of pyrene was kept constant at 2 µM, while that of the SAP conjugate was ranging between 0.0 and 1.2 mM. After an excitation at 330 nm, change in the ratios of fluorescence emission intensity at 370 nm (*I*_*1*_) and 380 nm (*I*_*3*_), started to occur at 0.13 mM, indicating the critical micelle concentration (CMC) of SAP conjugate

To calculate the critical micelle concentration (CMC) of the conjugate, the fluorescence spectrum of pyrene was used. The intensity ratio of the first and third fluorescence emission peaks of pyrene is very sensitive to aggregation of pyrene, and is indicative of the formation of micelles. An obvious trend change in intensity ratio of the first and third fluorescence emission peaks of pyrene, found at 0.13 mM, confirmed the formation of micelles (Fig. 2).

**Fig. 3:**
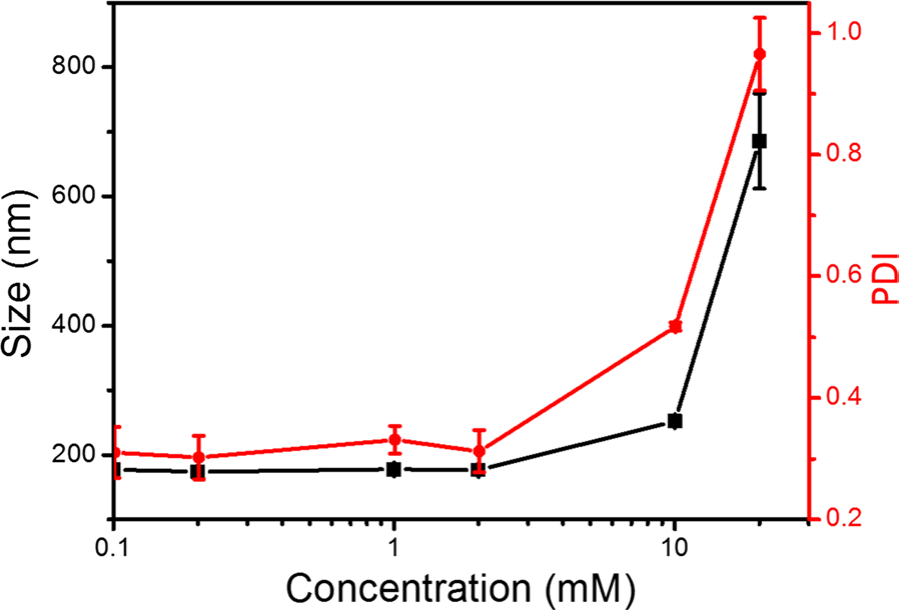
Particle size and polydistribution index (PDI) of SAP conjugate aggregates in water versus its concentration. The particle size of the SAP conjugate is around 180 nm when the concentration of SAP is less than 2 mM and the corresponding PDI is 0.3; represents a narrow distribution (or monodisperse). With increase in the concentration of SAP beyond 2 mM, the particle size of SAP conjugate quickly increased, suggesting a broad PDI (or polydisperse)

The SAP conjugate aggregated into small particles around 200 nm at low concentration in water and had a narrow polydispersity index (PDI) of 0.3. The SAP conjugate aggregated into bigger particles and had a wide PDI when the concentration of the conjugate was above 2 mM (Fig. 3). Finally, the aqueous solution of SAP conjugate lost fluidity and transited into a hydrogel when its concentration was higher than 20 mM.

**Fig. 4:**
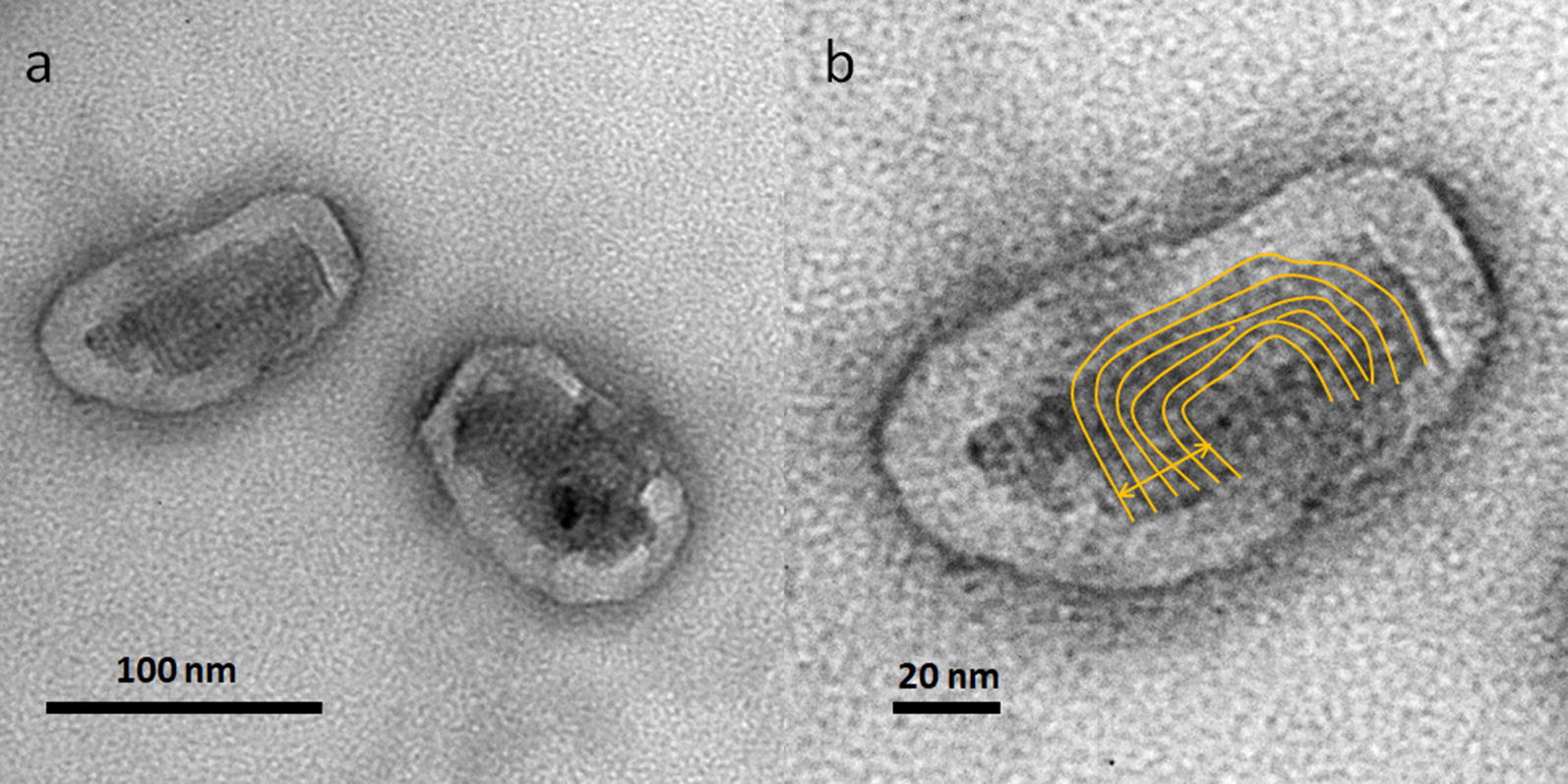
Morphology of SAP conjugates. **a** TEM image of SAP aggregates in water. **b** Magnified image showing the ordered nanostructure of the core where the distance between individual layers of the aggregates is 4 nm

Under TEM, SAP conjugate particles were calculated to be 82 ± 30 nm in length and 55 ± 17 nm in width (Fig. 4a and Additional file 6). It was evident that a disordered shell surrounded the ordered core while the ordered core of particles had a four nm sub-structure (Fig. 4b). The sub-structure size was in agreement with the diameter of the SAP conjugate indicating the sub-structure was formed by stacking of the SAP conjugate.

The contact angle (CA) measurement of the cast film of the SAP conjugate indicated that the SAP conjugate had excellent amphiphilicity and quickly adjusted to a new environment. For water, the CA of the SAP conjugate decreased from 21.8 ± 2.6° to 3.7 ± 1.8° in 4 min, indicating that the SAP conjugate had good hydrophilicity and capability to form a colloidal-stable dispersion in water. For oil, the CA was close to zero degrees demonstrating the super-lipophilic nature of SAP formulations and a potential to adsorb and interact with the hydrophobic wax layers on a leaf surface.

### Defining the phytotoxicity effect

**Fig. 5:**
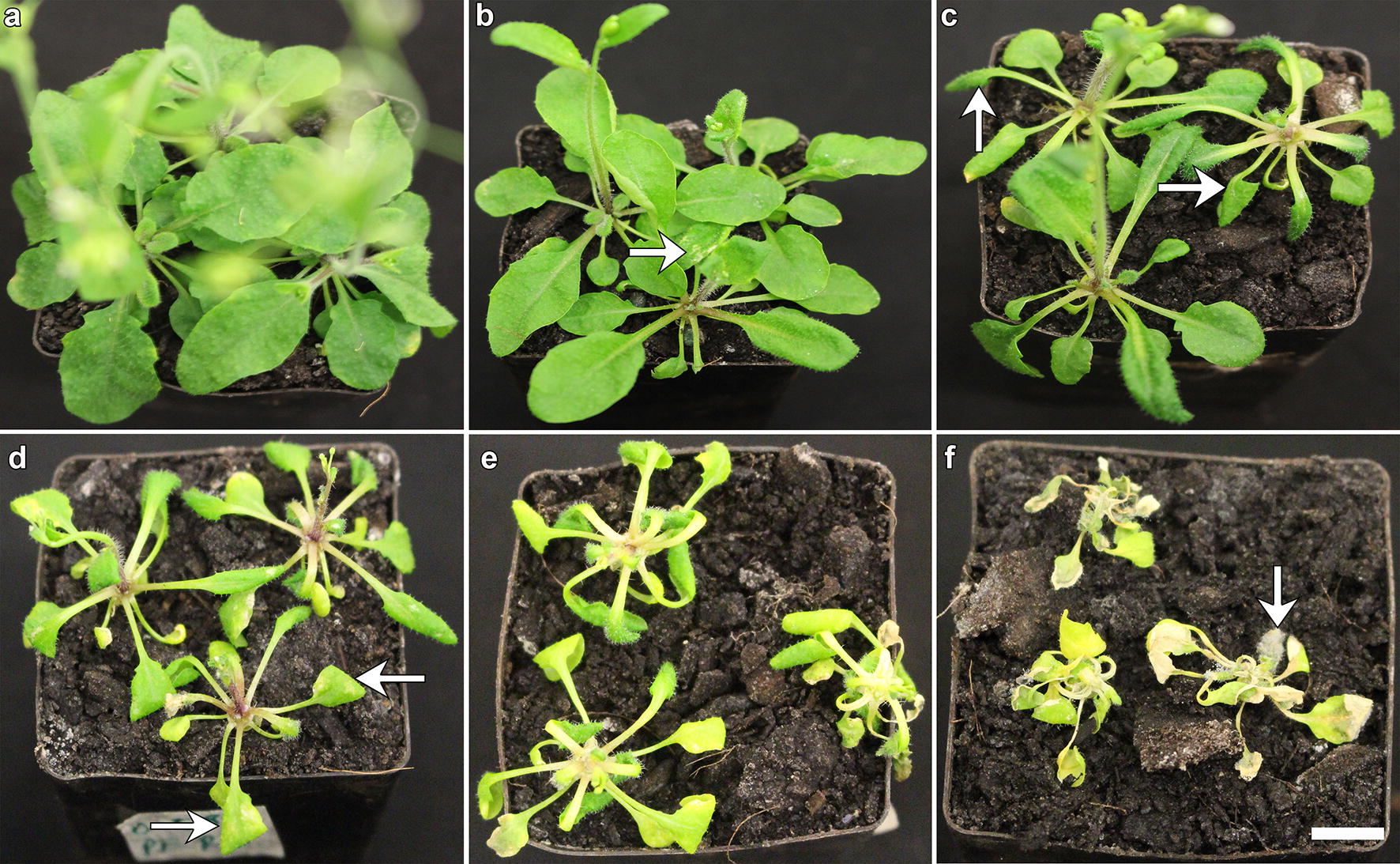
Quantification of herbicidal effect on a five-point scale. Three blind assessors independently ranked all the photographs of the plants on a five-point scale as described (n = 3 for each replicate). **a** 0 = healthy plant with no visible signs of leaf curling or necrosis. **b** 1 = minor leaf curling associated with or without slender tissue necrosis. **c** 2 = leaf curling associated with or without mosaic tissue necrosis. **d** 3 = prominent leaf curling associated with sizable tissue necrosis and chlorosis. **e** 4 = very prominent leaf curling along the axis, major necrosis and chlorosis. **f** 5 = pronounced leaf curling along the axis, major necrosis, chlorosis and death. Scale bar equals to one cm

To quantify the herbicidal effect after spray application of various formulations each collected image of the treated plants was assessed on a five-point scale ranging between 0 and 5 by blind assessment carried out by three independent assessors (n = 3 for each replicate). The 0–5 scale was as follows; 0 = healthy plant with no visible signs of leaf curling or necrosis (Fig. 5a); 1 = minor leaf curling associated with or without slender tissue necrosis (Fig. 5b); 2 = leaf curling associated with or without mosaic tissue necrosis (Fig. 5c); 3 = prominent leaf curling associated with sizable tissue necrosis and chlorosis (Fig. 5d); 4 = very prominent leaf curling along the axis, major necrosis and chlorosis (Fig. 5e); 5 = pronounced leaf curling along the axis, major necrosis, chlorosis and death (Fig. 5f).

### Effect of surfactant-based picloram delivery to *A. thaliana* plants

**Fig. 6:**
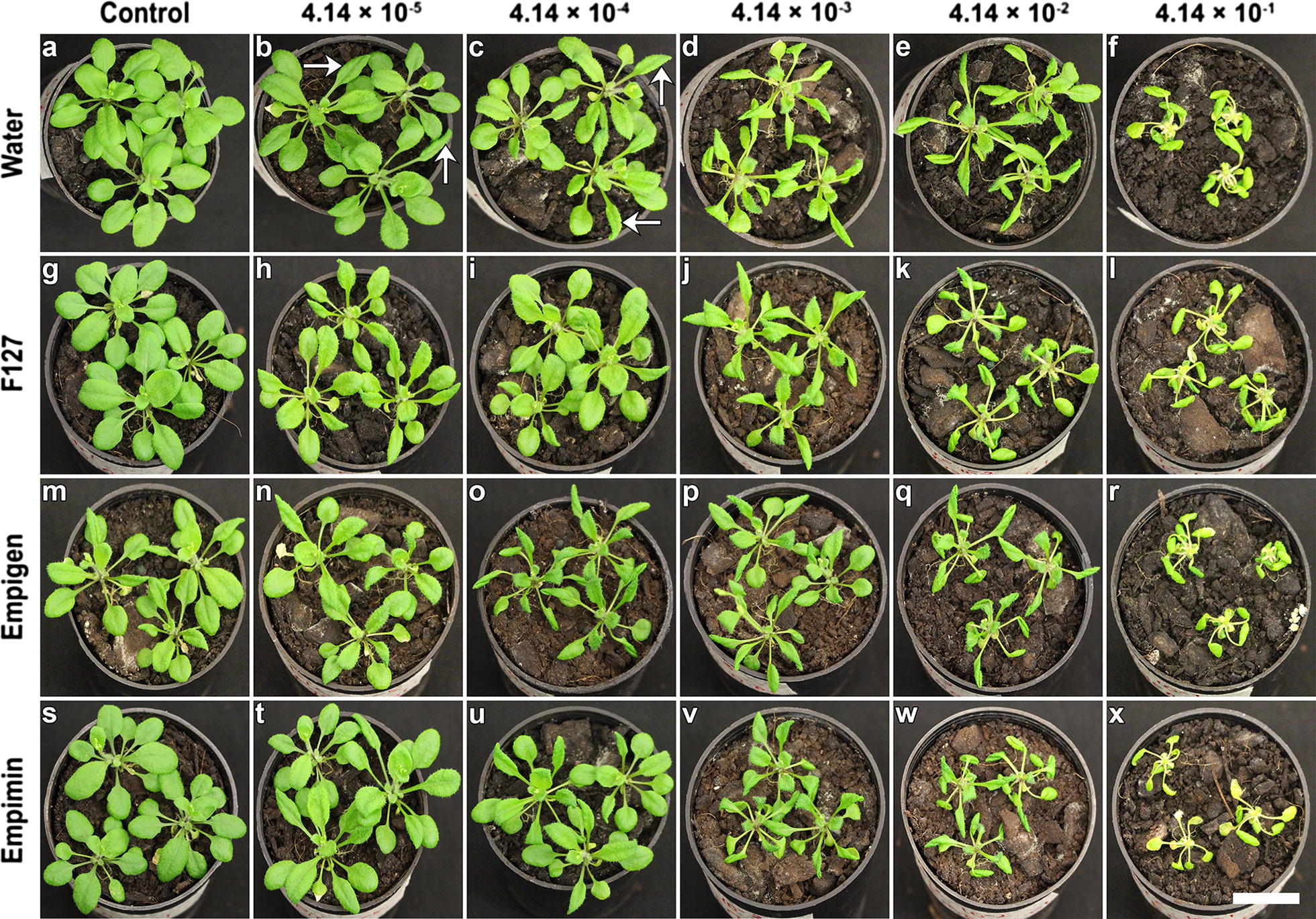
Surfactant based picloram delivery to *A. thaliana* plants. On the left hand side of the images given are the tested surfactants whose concentration was kept constant at 0.01% (w/v) while on the top of the images are the relevant concentrations of picloram ranging between 4.14 × 10^−5^ mM to 4.14 × 10^−1^ mM. **a** Water treated control *A. thaliana* plant 7 days after treatment. **b**–**f** Plants treated with increasing concentrations of picloram mixed in water. Arrows on **b** and **c** indicate the characteristic herbicidal effect of picloram leaf curling. From **d**–**f** the herbicidal effect elevated to a very severe leaf curling and a progressive increase in chlorosis was evident. Similar to the picloram in water treatments, plants spray applied with incremental doses of picloram mixed in F127, showed a gradual increase in phytotoxicity symptoms such as leaf curling, petiole curling and chlorosis from (**g**) to (**l**). **m**–**r** For a slow rise in the amount of picloram in empigen, the herbicidal effects was much stronger when compared to other similar treatments of the experiment. **s**–**x** For picloram delivered along with empimin the phytotoxicity impact was similar to F127 based herbicide delivery. Scale bar equals 1.5 cm

Picloram (mixed in water) applied to 3-week-old *A. thaliana* plants at different concentrations that ranged between 4.14 × 10^−5^ and 4.14 × 10^−1^ mM elicited a dose-dependent herbicidal effect (Additional file 7 and Fig. 6a–f). The herbicidal effect started with the occurrence of curled leaves that gradually became more pronounced to severe epinasty and chlorosis (Additional file 7 and Fig. 6a–f). A visually evident strong herbicidal effect, however, appeared only when the applied concentration of the picloram reached 4.14 × 10^−1^ mM. Similar to picloram in water treatments, the herbicidal effect of picloram when spray applied with F127 was also dose dependent and based on the visual symptoms, the minimal effective dose that killed the plants was determined to be 4.14 × 10^−1^ mM (Additional file 7 and Fig. 6g–l). Though picloram amalgamated with empigen elicited a slightly stronger herbicidal effect, the effective dose that killed the plants remained to be 4.14 × 10^−1^ mM (Additional file 7 and Fig. 6m–r). The herbicidal effect earlier observed for F127 and picloram in water treatments was observed for spray applications containing empimin and picloram (Additional file 7 and Fig. 6s–x).

**Fig. 7:**
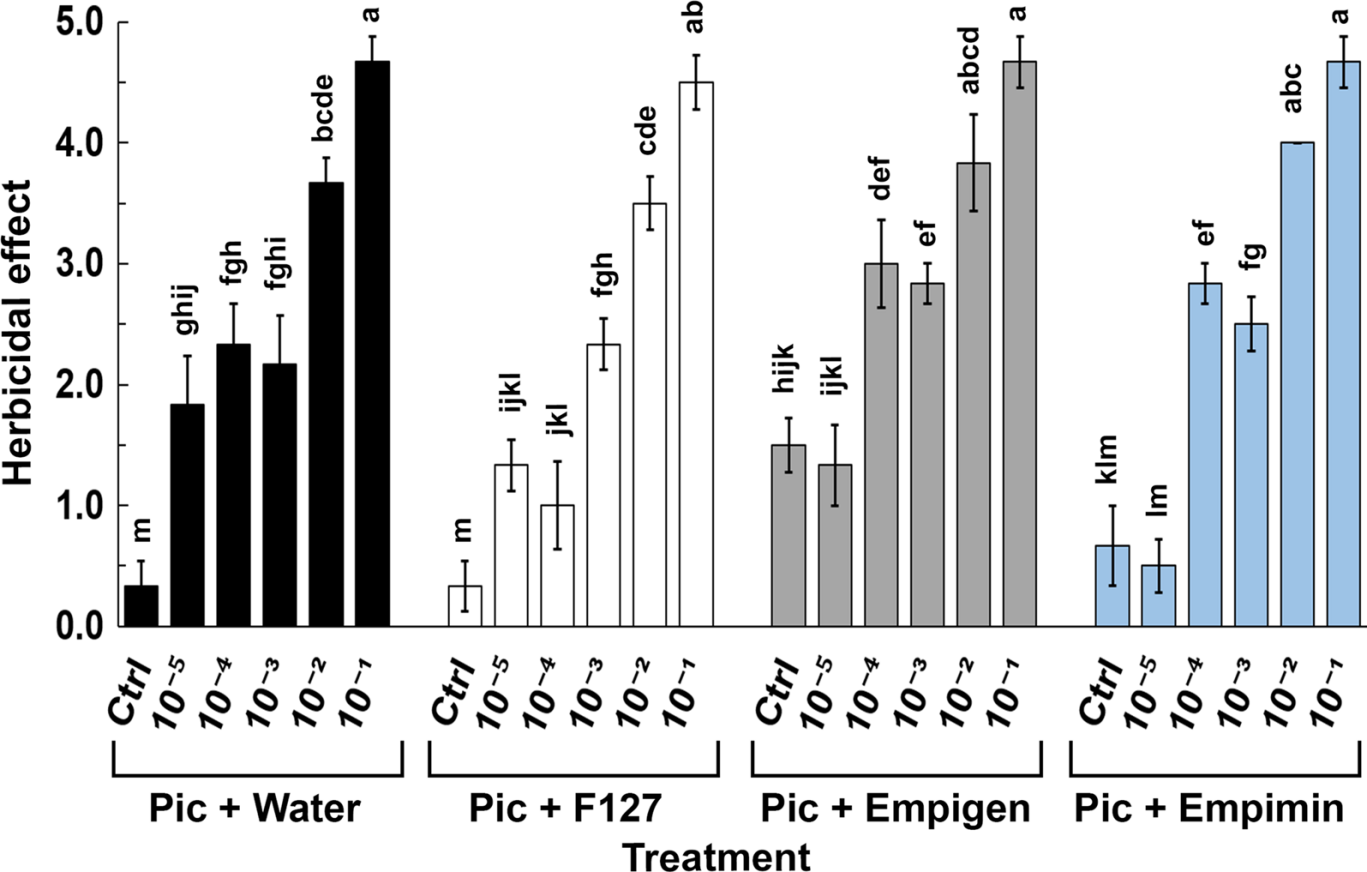
Herbicidal effect on *A. thaliana* following surfactant-assisted picloram delivery. For all treatments of picloram with water, F127, empimin and empigen, the highest concentrations 10^−2^ mM and 10^−1^ mM, (note multiply all x-axis concentrations by 4.14) showed the greatest herbicidal effect. Ctrl is the control for the relevant picloram solvents. Ctrl = control, Pic = picloram. The bars ± SE labelled with the same letters are statistically similar (p < 0.05), Duncan’s posthoc test

To quantify the herbicidal effect of the picloram spray applications, images were collected 3 days after spray application (Additional file 8). Except for treatment at 4.14 × 10^−3^ mM a dose-dependent trend in herbicidal effect was observed for picloram (in water) treatments between 4.14 × 10^−5^ mM and 4.14 × 10^−1^ mM. The herbicidal effect at a dose of 4.14 × 10^−1^ mM was statistically different from other picloram (in water) treatments (Additional file 8). For picloram administered with F127, doses of 4.14 × 10^−5^ mM and 4.14 × 10^−4^ mM did not show dose dependency but the effect was statistically different from those doses between 4.14 × 10^−3^ mM and 4.14 × 10^−1^ mM (Additional file 8). The overall herbicidal effect for individual treatments of empigen based picloram spray applications between 4.14 × 10^−5^ mM and 4.14 × 10^−1^ mM was very similar to that for picloram in water applications (Additional file 8). Though a dose-dependent trend was observed for picloram mixed with empimin, the herbicidal effect was statistically similar to other treatment groups (Additional file 8). For all the spray applied surfactant formulations after day seven, it was evident that 4.14 × 10^−1^ mM was the minimal effective dose that elicited a significant picloram-induced herbicidal effect (Fig. 7).

### Delivery of amphiphile–picloram (SAP) conjugate to *A. thaliana*

Based on the minimal dose of picloram required to kill *A. thaliana* plants the experiments related to SAP conjugate application used concentrations above 0.414 (4.14 × 10^−1^) mM. Before the spray application of the amphiphile–picloram (SAP) conjugate to the plants, its solubility was tested by mixing the SAP conjugate in water at concentrations of 0.585 (or 5.85 × 10^−1^) mM [equal to 0.05% (w/v)], 1.17 mM [0.1% (w/v)], 2.34 mM [0.2% (w/v)], 5.85 mM [0.5% (w/v)] and 11.70 mM [1.0% (w/v)]. The SAP conjugate formed an emulsion at all the concentrations tested while for picloram-oleyl (PO) and picloram (alone), a precipitate formed at the bottom of the tube at the same concentrations of that for the SAP conjugate (Additional file 9).

To assess the herbicidal efficacy, the SAP conjugate was mixed in water and spray-applied to 3-week-old *A. thaliana* plants (Additional file 10). With an increase in the concentration of spray-applied SAP to the plants, a clear dose dependency of the herbicidal effect was observed after 3 days (Additional file 10). For the spray application of SAP at a concentration of 0.58 mM the plants were observed to have a strong epinastic response (Additional file 10: Figure S10b), which further extended to petiole curling with an increase in applied SAP conjugate concentration to 1.17 mM (Additional file 10: Figure S10c). With a progressive increase of SAP conjugate concentration to 2.34 mM (Additional file 10: Figure S10d) curled leaves and petioles twisted around the axis of the plant which became more prominent with further increases in the concentration of applied SAP conjugate to 5.85 mM and 11.70 mM (Additional file 10: Figure S10e–f). PO application on the contrary, did not have the same phytotoxicity effect as that of SAP, when applied at the same concentrations. The leaf curling observed for PO spray applications was limited to the lamina, irrespective of the concentration of the PO applied (Additional file 10: Figure S10g–i). Picloram (in water) spray applications showed a consistent and strong herbicidal effect that was similar to the effect elicited by SAP conjugate applications. Unlike other treatments, picloram induced herbicidal effect on the plants was characterised by the presence of chlorosis even when applied at the lowest concentration of 0.58 mM and severe tissue necrosis was observed at high concentrations of 5.85 mM and 11.70 mM (Additional file 10: Figure S10m–r).

**Fig. 8:**
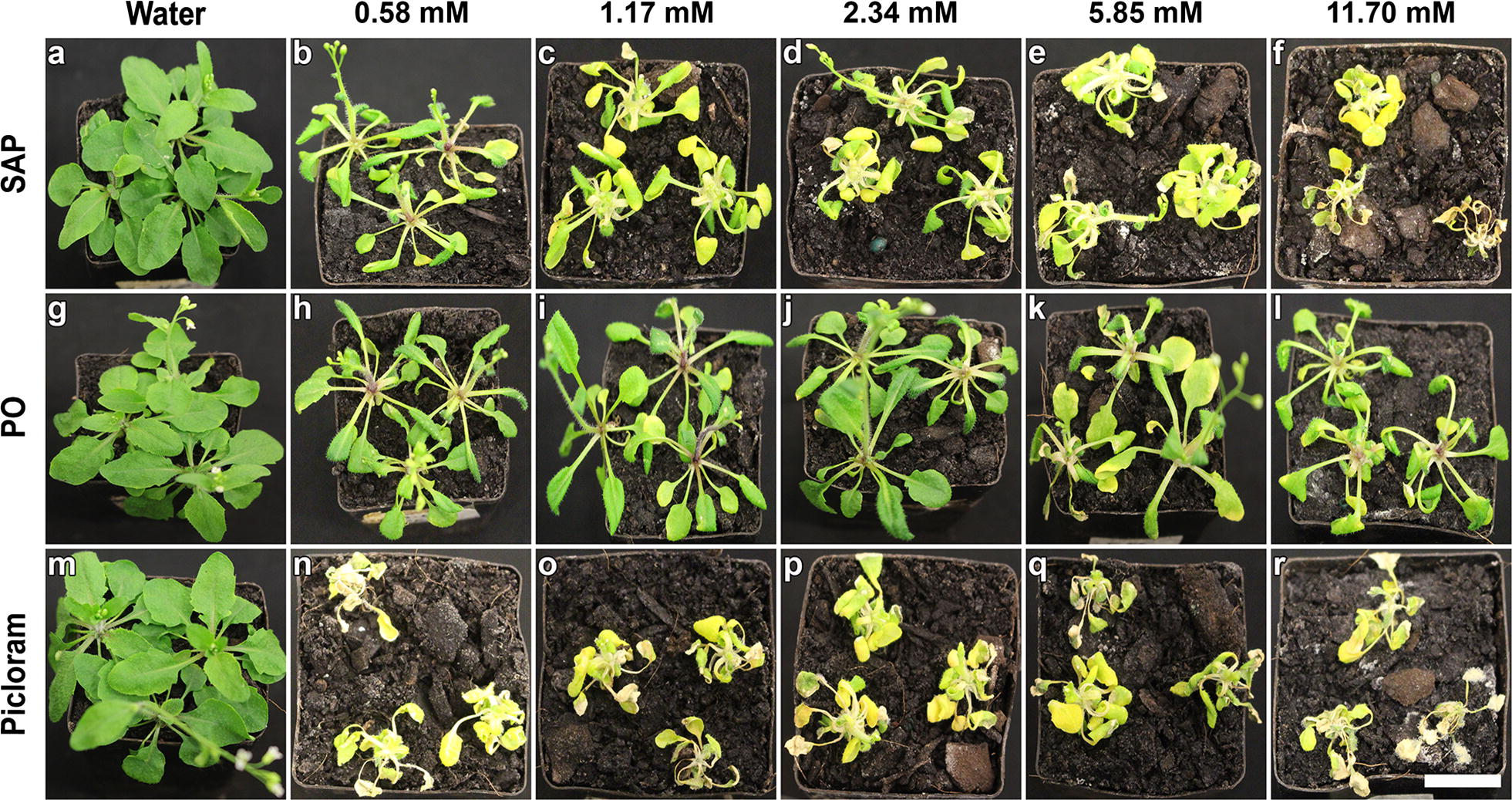
Herbicidal impact on plants post SAP treatments. **a** Control plants are imaged 7 days after the water treatment. **b** For 0.58 mM SAP application induced phytotoxicity on plants defined by leaf and petiole curling, stunted stem growth and chlorosis. **c** With further increase in the dosage of SAP to 1.17 mM, the effects of phytotoxicity elevated to the tissue necrosis. For a gradual increase in the dosage from **d** 2.34 mM through **e** 5.85 mM to **f** 11.70 mM the herbicidal impact also respectively intensified leading plant to death. When compared to water treated control plants (**g**), PO treated plants with 0.58 mM (**h**), 1.17 mM (**i**) and 2.34 mM (**j**) doses, developed epinasty. On the contrary, PO treated plants 5.85 mM (**k**) and 11.70 mM (**l**) exhibited chlorosis along with epinasty. Except for the water treated control plants (**m**), all the picloram treatments elicited an intense leaf curling, chlorosis and tissue decay in a dose-dependent manner (**n**–**r**). Scale bar equal to 1 cm

Juxtaposed with water treated controls, the herbicidal effect 7 days after the spray application of SAP conjugate intensified on *A. thaliana* plants in a dose-dependent manner (Fig. 8a). Plants exposed to 0.58 mM SAP exhibited stunted stem growth, chlorosis and tissue necrosis, in addition to the earlier observed epinasty and petiole curling effects (Fig. 8b). These effects further escalated with increase in the concentration of SAP conjugate from 1.17 mM through to 11.70 mM, ultimately leading to plant death (Fig. 8c–f). Across the range of the tested concentrations, a slight increase in the herbicidal effect was detected 7 days after the PO spray treatments (Fig. 8g–l). The leaf curling observed on day three progressed to petiole curling and chlorosis only when the concentration of applied PO was raised to either 5.85 mM or 11.70 mM (Fig. 8k–l). For plants exposed to picloram (in water) formulations, plant death was observed from the tested lowest concentration (0.58 mM) through to the highest concentration (11.70 mM). Consistently across all the treatments extensive leaf curling, severe chlorosis and tissue necrosis were identified (Fig. 8m–r).

### Quantification of the herbicidal effects of SAP conjugate spray applications

**Fig. 9:**
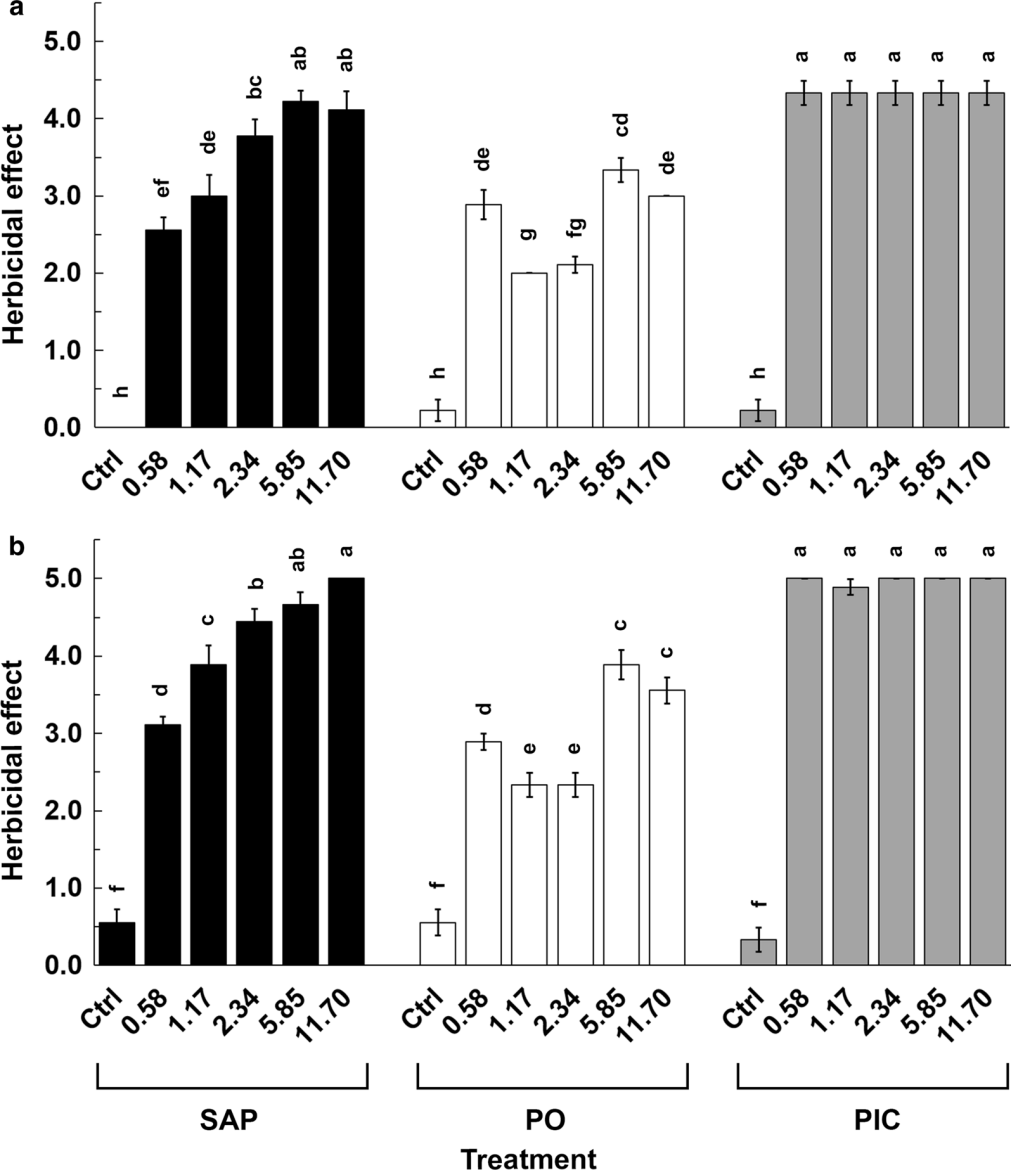
Quantification of herbicidal effect after day three and seven SAP spray applications. **a** After the SAP spray applications, on the third day, the herbicidal effect followed a dose dependent trend and the applications between doses 2.34 and 11.70 are statistically similar. PO spray applications elicited an inconsistent herbicidal effect on treated *A. thaliana* plants. Picloram (PIC) spray applications elicited an equal and statistically similar herbicidal effect. **b** The effect of herbicide treatment at day seven was similar to day three except the intensity of the effect was increased. Bars ± SE labelled with same letters are statistically similar (p < 0.05) Duncan’s posthoc test performed separately on (**a**) and (**b**)

SAP conjugate spray applications elicited a dose-dependent herbicidal effect as observed on day three. For the lowest dose of SAP conjugate applied (0.58 mM), the herbicidal effect recorded was 2.56 ± 0.17 which then gradually increased to 4.11 ± 0.25 with each increment in the applied dosage of SAP to 11.70 mM (Fig. 9a). The herbicidal effect recorded for 5.85 mM and 11.70 mM SAP conjugate applications was statistically similar to that of picloram spray applications. Spray application of PO to plants at equal concentrations, did not follow any noticeable trend for the observed herbicidal effect. On the other hand, an equal and statistically similar effect was found for all picloram concentrations between 0.58 and 11.70 mM (Fig. 9a).

Phytotoxicity observations at 7 days after spray application of SAP conjugate, PO and picloram were broadly consistent with the day three observations (Fig. 9b). There was a dose-dependent trend of increasing herbicidal effect for all concentrations of SAP conjugate tested and at 5.85 mM and above, the effect was the same as that induced by picloram (in water) alone (Fig. 9b). For PO at both time points, there was not a clear dose-dependent response. The herbicidal effect elicited by picloram (in water) application was the same for all the tested concentrations (Fig. 9b).

## Discussion

The use of self-assembling lipids for the delivery of therapeutics such as antibiotics [28] and anti-cancer drugs [29] is well known in the pharmaceutical industry but their use for the delivery of agrochemical actives such as herbicides, has not been examined. Here, we have described the synthesis of a novel self-assembling amphiphile–picloram conjugate for the specific delivery of agrochemicals that aligns with the drive for green chemistry and sustainable agricultural practices. We have shown that when a plant such as *A. thaliana* grown in vitro was sprayed with agriculturally relevant concentrations of the SAP conjugate containing the herbicide picloram that the nanoformulation was as equally effective for killing target plants as current surfactant-based formulations. This approach to agrochemical delivery has several clear advantages over surfactant-based delivery that include the prevention of crop and off target toxicity, reduction in chemical load and use of less water which, together, benefits the environment and has the potential to increase crop productivity. The three radiating arms that formed the SAP conjugate were the key features of this nanomaterial that enabled the conjugate to form a stable emulsion in water and, when applied, to interact directly with the target hydrophobic plant leaf surface.

Picloram has a pKa value of 2.30 and is poorly soluble in water at 430 mg per litre [30]. Surfactants and adjuvants are therefore tank mixed to influence the physicochemical properties of the agrochemical active and thereby enhance the uptake of the active by the plants [31]. Surfactants (including pluronic stabilisers) have been described several times as the cause of irreversible damage to plants that ultimately lead to yield loss but surfactants are also toxic to the environment and are detrimental to human health [32]. To overcome these disadvantages associated with surfactant-based agrochemical delivery we have earlier proposed the use of LC systems for the safe delivery of agrochemicals to plants [17]. Phase structure is often sterically stabilised by the use of pluronic stabilisers and the physiochemical properties of the LC systems are the critical rate limiting factors that influence their interaction with hydrophobic plant surfaces [33, 34]. Similarly, linear agrochemical conjugates synthesised by conjugating picloram to lipids (phytantriol and glyceryl monooleate) and that attain higher loading efficiency of picloram are limited in their use as an agrochemical active due to the presence of a stearic stabiliser [8].

**Fig. 10:**
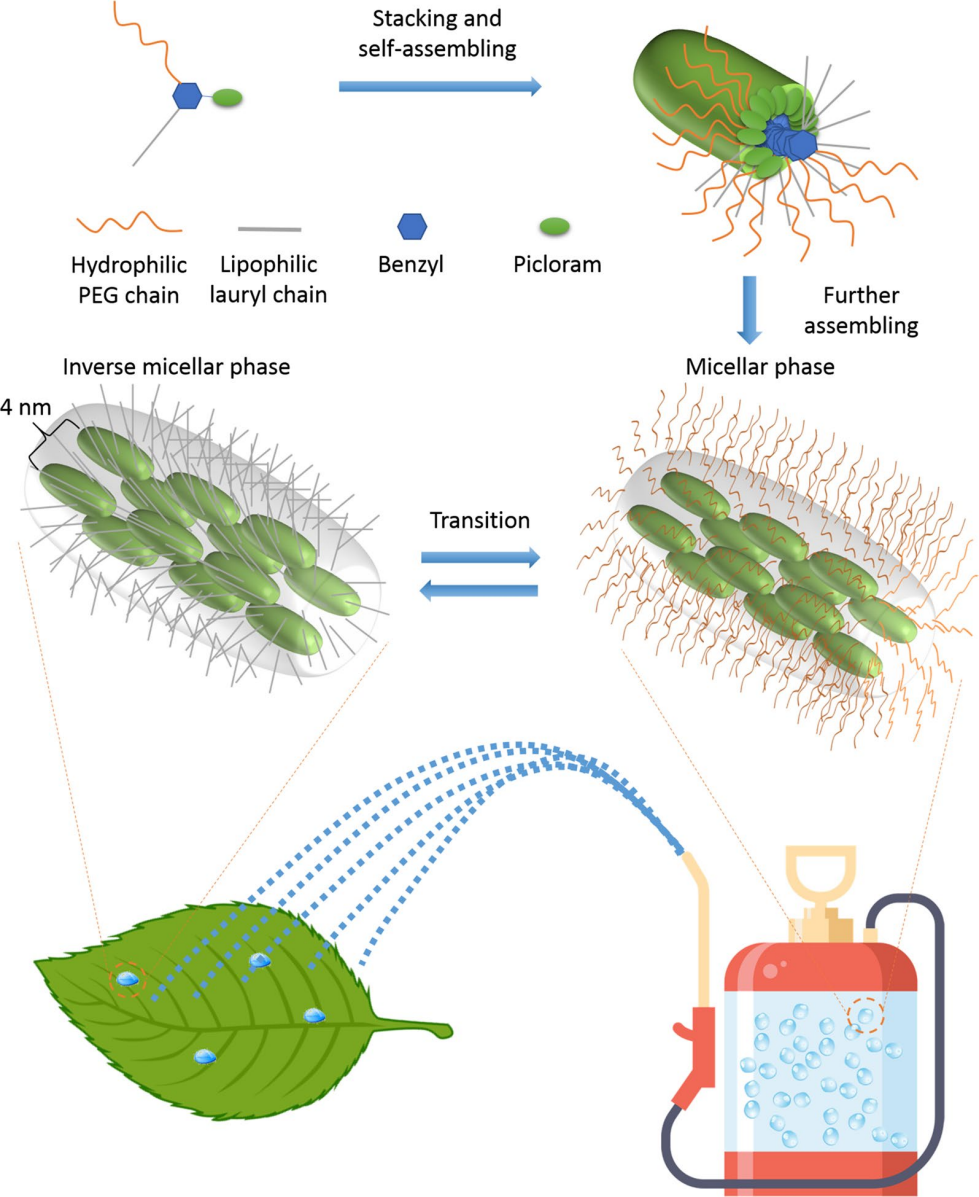
Schematic representation of the SAP conjugate. The SAP has three extending arms made up of a hydrophilic polyethelene glycol (PEG) chain, a lipophilic lauryl chain and an amphiphobic agrochemical active. Green cylindroids represent the self-assembling SAP conjugates. In water, SAP self-assembles to a micellar phase, which when spray applied to plant leaf surfaces and upon the evaporation of water, quickly reorients to form an inverse micellar phase. Extending arms of SAP promote easy transition between the phases and help maintain the colloidal stability of the formulation. With an intact four nm internal nano-structure (indicated by spacing between cylindroids), SAP strongly adsorbs on to hydrophobic surfaces even in the absence of surfactants

Therefore, we synthesised SAP conjugate whose arms reduce the free energy barrier and help its aqueous formulation to attain colloidal stability, leading to a quick transition between micellar and inverse micellar structures either in a hydrophilic or lipophilic environment respectively. The low water contact angle of the SAP conjugate provided a very good indication of the hydrophobic switch and the natural ability of the conjugate to attain better colloidal stability [35]. Such a stable aqueous formulation of the SAP conjugate was shown to retain its four-nanometer internal lamellar nano-structure when viewed using TEM. The ordered internal nano-structure of the SAP aggregates was also indicated by pyrene peak intensities beyond 0.7, while PDI and a CMC plateau phase were suggestive of larger SAP aggregates. It is plausible that freely transitioning SAP conjugate moieties make up the disordered outer shell and further contribute to the colloidal stability of the formulation. We propose that the ordered internal structure facilitates better interaction with, and without damage to, the plant hydrophobic epicuticular wax layer [12, 33] (Fig. 10). Surfactant-based agrochemicals, however, do not interact with the epicuticular wax layer but act by solubilising the wax layer and thus damaging it irreversibly [17].

When the aqueous formulation of the self-assembling conjugate was spray applied to hydrophobic leaf surfaces, the extending arms of the SAP conjugate enabled its quick transition upon the evaporation of the water. For the activity of the SAP conjugate, it is inaccurate to assume that herbicidal activity will occur after the disassociation of picloram from the conjugate. We predict that the entire SAP conjugate is adsorbed onto the plant cuticular surface. Picloram, because of its strong covalent bond with 3-(dodecyloxy)-5-(EO_7_)phenol (component 5) is very unlikely to freely disassociate from the conjugate. After adsorption the SAP conjugate acts like a lipophilic non-electrolyte in that it diffuses through the amorphous regions of the cuticle to elicit the herbicidal effect [36]. The herbicidal effect is thus a constitutive effect of the exposed active site of the picloram molecule that is undisturbed during the covalent bonding with 3-(dodecyloxy)-5-(EO_7_) phenol.

*Arabidopsis thaliana* plants elicited a dose-dependent herbicidal effect when treated with increasing doses of picloram that contained equal amounts of either of the surfactants empimin, empigen or F127. To mimic the current agricultural practices, the laboratory experiments included agriculturally relevant doses of picloram and surfactants. Surfactants are conventionally tank mixed with agrochemical actives such as picloram to enhance the herbicidal effect of the active, by influencing the physiochemical properties of both the plant cuticle and the active ingredient [37, 38]. Despite the addition of the surfactants, picloram applications failed to induce a statistically significant herbicidal effect for the range of picloram concentrations that we tested, and within the course of the experiments. Therefore, 4.14 × 10^−1^ mM was determined to be the minimal effective dose of picloram that completely killed *A. thaliana*. Matching the surfactant or adjuvant with the active ingredient is a fine art and is necessary for selection of the combination of surfactant and active that enables the active exert its greatest effect.

Picloram like 2,4-D is an amphiphobic phenoxy herbicides (or auxin mimic) that is routinely used to eliminate dicot weeds such as wild radish (*Raphanus raphanistrum*), wild mustard (*Sinapis arvensis*) and wild turnip (*Myagrum rugosum*) in monocot crops such as wheat (*Triticum aestivum*), barley (*Hordeum vulgare*) and oats (*Avena sativa*) [39]. Epinasty, tissue necrosis, chlorosis and plant death characterise the herbicidal effect elicited by phenoxy herbicides [12]. Auxin mimics act by disrupting the hormonal homeostasis and thereby induce tissue necrosis; inducing over-production of ethylene, reactive oxygen species and hydrogen cyanide; causing growth inhibition and reduction in CO_2_ assimilation and that ultimately leads to plant death [40, 41].

In our laboratory experiments, for all the formulations, a dose range that was agriculturally relevant was tested. It is likely that the diffusion of SAP conjugate is slower than picloram by itself due to its larger size that in turn limits the rate of diffusion of the conjugate into the amorphous regions of the cuticle, that are usually available for the diffusion of lipophilic non-electrolytes [36]. At the tested doses, picloram oleyl failed to exhibit any significant herbicidal effect compared with that shown for SAP conjugate or picloram formulations. The lack of herbicidal effect indicated that alkylation of an amphiphobic agrochemical active, such as picloram, does not enhance efficacy.

## Conclusions

It was recently shown that agrochemicals, such as pesticides, contribute to the largest fraction of volatile organic compound sources that not only damage the environment but also increasingly impact human health [5]. In addition, surfactant based agrochemicals irreversibly damage plant cuticular surfaces [3, 42]. To overcome the deleterious effects posed by traditional agrochemical formulations we propose the use of surfactant-free conjugate-based self-assembly formulations. We expect that the interactions of self-assembling lipid-based conjugates with the hydrophobic surface of crop plant leaves will, as has been found previously for at least one other lipid based nano-formulation, not damage the epicuticular wax micromorphology therefore reducing both phytotoxicity and potential yield loss [12]. Use of self-assembling formulations can also prevent off-target environmental impacts by minimising off-target toxicity, require less water for spray application and the amount of agrochemical runoff will be reduced [17]. Key to understanding how agrochemical formulations penetrate into plant cells is in understanding the ultrastructure of the cuticle on plant surfaces which is characterised by the presence of cuticular waxes that are interspersed in a cutin matrix [43, 44]. Here, we have used the model plant *A. thaliana* in our proof of concept study but clearly agriculturally relevant crop and weed species must now be examined for their interactions with self-assembling conjugates and with a variety of actives. We expect that self-assembling conjugates will find broad use in agriculture for the delivery of actives such as herbicides, pesticides and fungicides and that their use will strengthen existing practises for sustainable agriculture.

## Additional files

**Additional file 1: Figure S1.** Component (2) 3-(dodecyloxy)-5-(hydroxymethyl)phenol. ^1^H NMR (400 MHz, CDCl_3_, δ): 0.79–0.82 (t, 3H, –CH_3_); 1.15–1.26 (m, 16H, –CH_2_–); 1.30–1.37 (m, 2H, –CH_2_–); 1.66–1.73 (m, 2H, –CH_2_–); 3.77–3.81 (t, 2H, –CH_2_–); 4.46 (s, 2H, –CH_2_–); 6.24–6.25 (t, 1H, –C_6_H_3_–); 6.32–6.33 (t, 1H, –C_6_H_3_–); 6.35–6.36 (t, 1H, –C_6_H_3_–); 7.89 (s, 0.5H, –OH).

**Additional file 2: Figure S2.** Component (4) Ots-EO_7_-Me. ^1^H NMR (400 MHz, CDCl_3_, δ): 2.37 (s, 3H, –CH_3_); 3.3 (s, 3H, –OCH_3_); 3.46–3.48 (t, 2H, –CH_2_–); 3.5 (s, 4H, –CH_2_–); 3.54–3.58 (m, 20H, –CH_2_–); 3.6–3.62 (t, 2H, –CH_2_–); 4.07–4.09 (m, 2H, –CH_2_–); 7.26–7.28 (d, 2H, –C_6_H_4_–); 7.71–7.73 (d, 2H, –C_6_H_4_–). The ^1^H NMR spectrum of component (4) Ots-EO_7_-Me.

**Additional file 3: Figure S3.** Component (5) 3-(dodecyloxy)-5-(EO_7_)phenol. ^1^H NMR (400 MHz, CDCl_3_, δ): 0.86–0.9 (t, 3H, –CH_3_); 1.26–1.35 (m, 16H, –CH_2_–); 1.39–1.46 (m, 2H, –CH_2_–); 1.72–1.79 (m, 2H, –CH_2_–); 3.37 (s, 3H, –CH_3_); 3.53–3.55 (m, 2H, –CH_2_–); 3.63–3.68 (m, 28H, –CH_2_–); 3.69–3.72 (m, 2H, –CH_2_–); 3.82–3.85 (t, 2H, –CH_2_–); 3.9–3.93 (t, 2H, –CH_2_–); 4.1–4.12 (t, 2H, –CH_2_–); 4.6 (s, 2H, –CH_2_–); 6.37–6.38 (t, 1H, –C_6_H_3_–); 6.51–6.53 (d, 2H, –C_6_H_3_–). The ^1^H NMR spectrum of component (5) 3-(dodecyloxy)-5-(EO_7_)phenol.

**Additional file 4: Figure S4.** Component (6) 3-(dodecyloxy)-5-(EO_7_)benzyl-PIC. ^1^H NMR (400 MHz, CDCl_3_, δ): 0.85–0.89 (t, 3H, –CH_3_); 1.25–1.34 (m, 16H, –CH_2_–); 1.38–1.45 (m, 2H, –CH_2_–); 1.71–1.78 (m, 2H, –CH_2_–); 3.36 (s, 3H, –CH_3_); 3.52–3.55 (m, 2H, –CH_2_–); 3.63–3.68 (m, 4H, –CH_2_–); 3.71–3.73 (m, 2H, –CH_2_–); 3.82–3.84 (t, 2H, –CH_2_–); 3.89–3.92 (t, 2H, –CH_2_–); 4.09–4.11 (t, 2H, –CH_2_–); 5.31 (s, 2H, –CH_2_–); 5.53 (s, 2H, –NH_2_); 6.41–6.42 (t, 1H, –C_6_H_3_–); 6.56–6.57 (d, 2H, –C_6_H_3_–). The ^1^H NMR spectrum of component (6) 3-(dodecyloxy)-5-(EO_7_)benzyl-PIC.

**Additional file 5: Figure S5.** Component (6) 3-(dodecyloxy)-5-(EO_7_)benzyl-PIC. ^13^C NMR (400 MHz, CDCl_3_, δ): 14.13 (s); 22.68 (s); 26.03 (s); 29.21 (s); 29.34–29.39 (d); 29.58–29.66 (m); 31.91 (s); 59.02 (s); 67.49–68.12 (t); 69.63 (s); 70.49–70.79 (t); 71.91 (s); 76.79–77.42 (t); 101.57 (s); 106.32–106.83 (d); 114.06–114.94 (d); 137.06 (s); 144.39 (s); 146.74 (s); 148.96 (s); 160.04–160.41 (d); 163.33 (s).

**Additional file 6: Figure S6.** Size distribution of the SAP molecules. From the TEM images the length and width of SAP particle was estimated to be 82 ± 30 nm and 55 ± 17 nm respectively. Though TEM images are an indicative of smaller SAP conjugates, a through sampling regime can confirm the larger size of the aggregates.

**Additional file 7: Figure S7.** Surfactant-assisted delivery of picloram to *A. thaliana*. The image on the left hand side is labelled with relevant surfactant whose concentration was kept constant at 0.01% (w/v) while the respective concentrations of picloram were given on the top. (a) Control *A. thaliana* plant three days after water treatment. (b to f) The herbicidal effect intensified with the increase in the concentration of picloram (mixed in water) spray application and are marked by the presence of leaf curling (c to f), chlorosis (d to f) and petiole curling (f). (g to l) F127 based picloram treatments were similar to picloram (in water) treatments. (m to r) A stronger phytotoxicity effect on images (m to r) was evident for the treatments containing incremental doses of picloram in empigen. (s to x) For picloram delivered with empimin the herbicidal effect was similar to the same effect earlier observed for F127 based picloram delivery. Scale bar approximately equals to 1.5 cm.

**Additional file 8: Figure S8.** Herbicidal effect on *A. thaliana* after surfactant assisted picloram delivery. Three days after the application of picloram separately with water, F127, empimin and empigen, maximum herbicidal effect was observed for the highest concentration of picloram (10^−1^ mM) applied. Note that in the figure all x-axis concentrations are multiplied by 4.14, Ctrl is the control for the relevant picloram solvents. Ctrl = control, Pic = picloram. The bars ± SE labelled with the same letters are statistically similar (p < 0.05), Duncan’s posthoc test.

**Additional file 9: Figure S9.** Solubility of SAP, PO and picloram in water. Self-assembly picloram when mixed in water formed a cloudy emulsion while PO and picloram at similar concentrations formed a precipitate.

**Additional file 10: Figure S10.** Effects of SAP delivery to plants. The images are labelled with relevant formulations on the left hand side with which they were spray applied while on the top are their respective concentrations. (a) Water treated healthy control plant three days after the spray treatments. The herbicidal effect of the SAP on plants was evident by the presence of leaf curling that elevated to petiole curling with the increase in dosage of SAP from (b) 0.58 mM through (c) 1.17 mM to (d) 2.34 mM. In addition to leaf and petiole curling, further increase in the concentration of SAP from 2.34 mM to (e) 5.85 mM and (f) 11.70 mM caused the leaves to twist along the main axis of the plant. (g) Water treated control for PO treatments. For PO applications at low concentrations, the phytotoxicity was limited to leaf curling (h to l) irrespective of the concentration. (m) *A. thaliana* plant treated with water. On the contrary, picloram applications induced a strong phytotoxicity marked by (n to p) the presence of chlorosis, petiole and leaf curling, (q and r) tissue necrosis and death. Scale bar equal to one cm.
