# Supplementary figures and images for: Novel self-assembling conjugates as vectors for agrochemical delivery
Source: J Nanobiotechnology. 2018 Nov 21;16:94. doi: 10.1186/s12951-018-0423-5 (PMC6247628; doi:10.1186/s12951-018-0423-5)

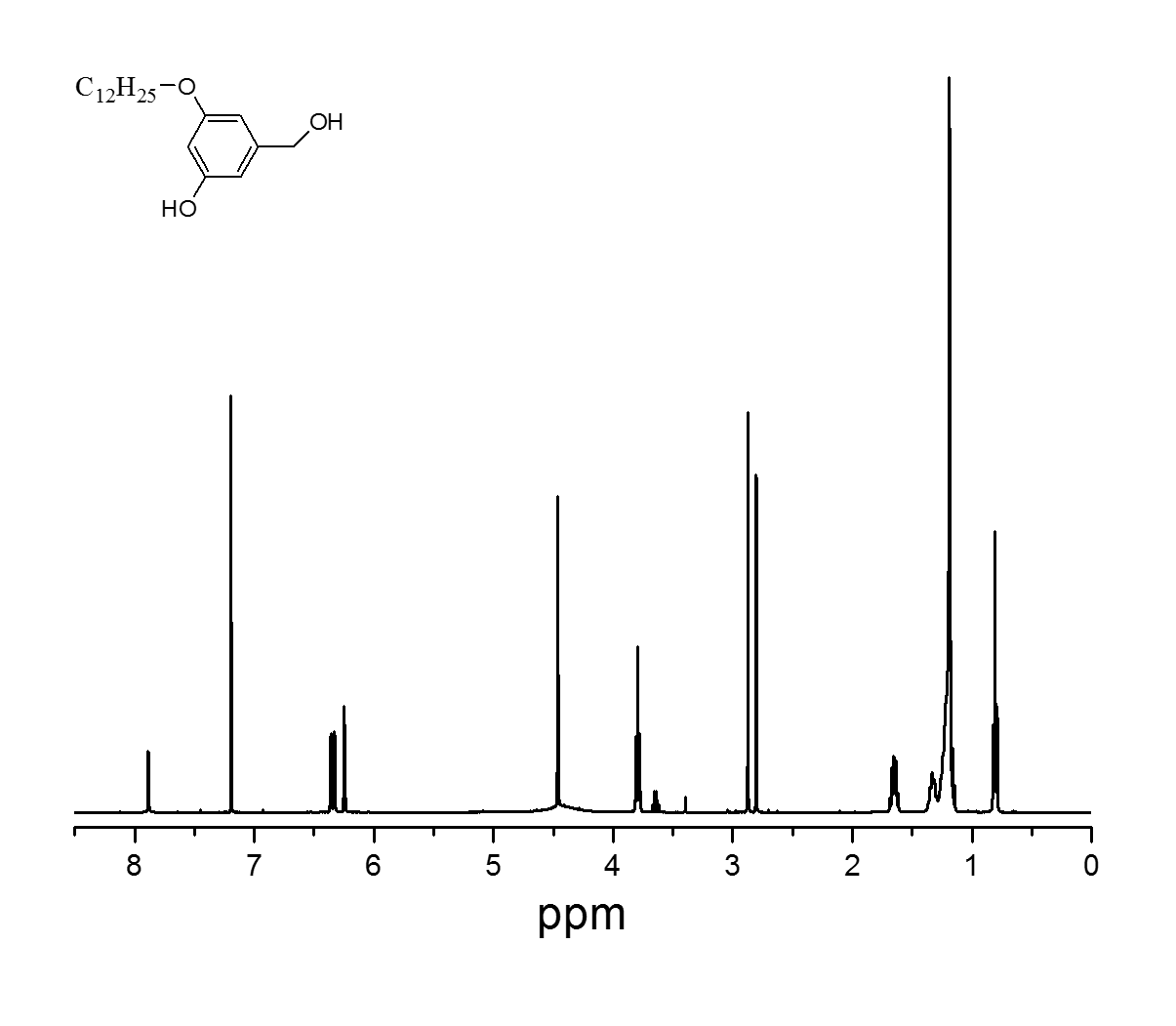

Supplement: Supplementary file 1 — Additional file 1: Figure S1. Component (2) 3-(dodecyloxy)-5-(hydroxymethyl)phenol. 1H NMR (400 MHz, CDCl3, δ): 0.79–0.82 (t, 3H, –CH3); 1.15–1.26 (m, 16H, –CH2–); 1.30–1.37 (m, 2H, –CH2–); 1.66–1.73 (m, 2H, –CH2–); 3.77–3.81 (t, 2H, –CH2–); 4.46 (s, 2H, –CH2–); 6.24–6.25 (t, 1H, –C6H3–); 6.32–6.33 (t, 1H, –C6H3–); 6.35–6.36 (t, 1H, –C6H3–); 7.89 (s, 0.5H, –OH). [file 12951_2018_423_MOESM1_ESM.tif]

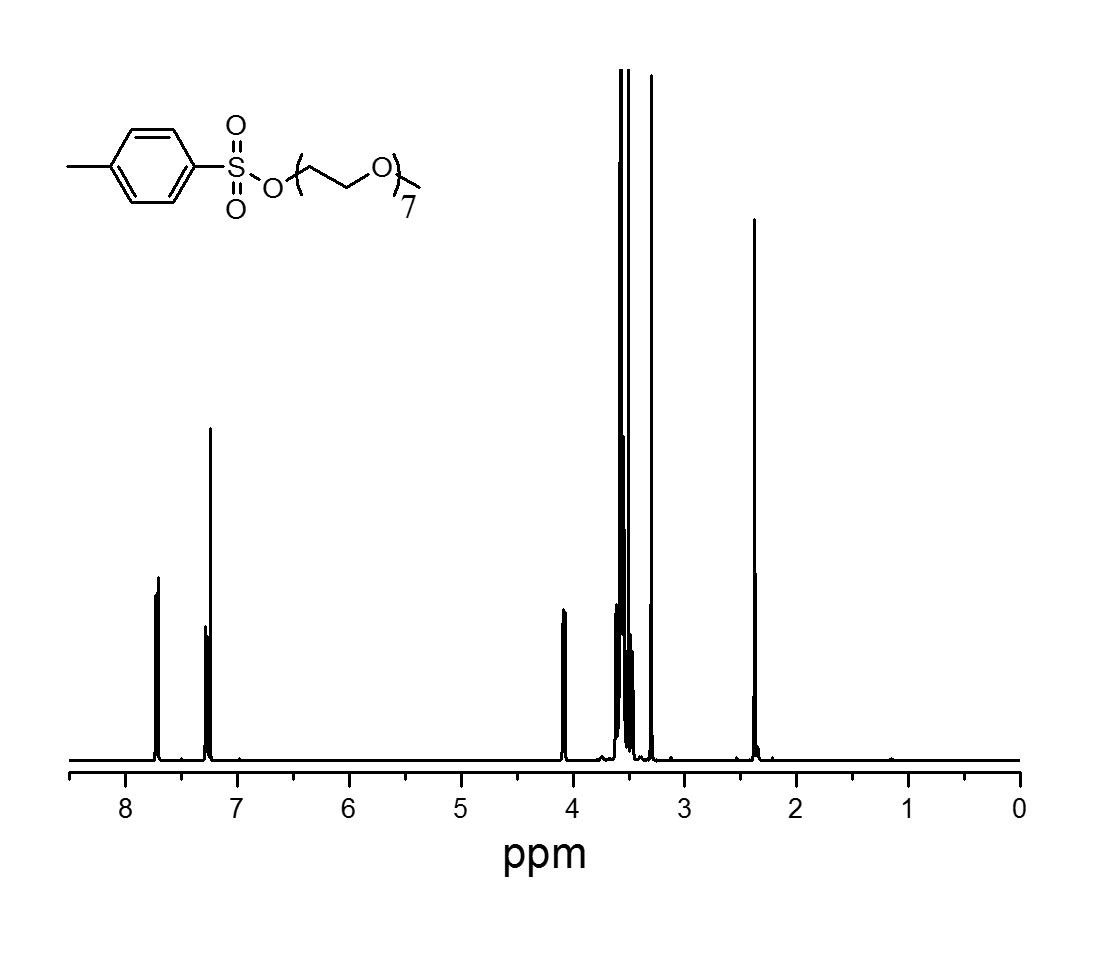

Supplement: Supplementary file 2 — Additional file 2: Figure S2. Component (4) Ots-EO7-Me. 1H NMR (400 MHz, CDCl3, δ): 2.37 (s, 3H, –CH3); 3.3 (s, 3H, –OCH3); 3.46–3.48 (t, 2H, –CH2–); 3.5 (s, 4H, –CH2–); 3.54–3.58 (m, 20H, –CH2–); 3.6–3.62 (t, 2H, –CH2–); 4.07–4.09 (m, 2H, –CH2–); 7.26–7.28 (d, 2H, –C6H4–); 7.71–7.73 (d, 2H, –C6H4–). The 1H NMR spectrum of component (4) Ots-EO7-Me. [file 12951_2018_423_MOESM2_ESM.tif]

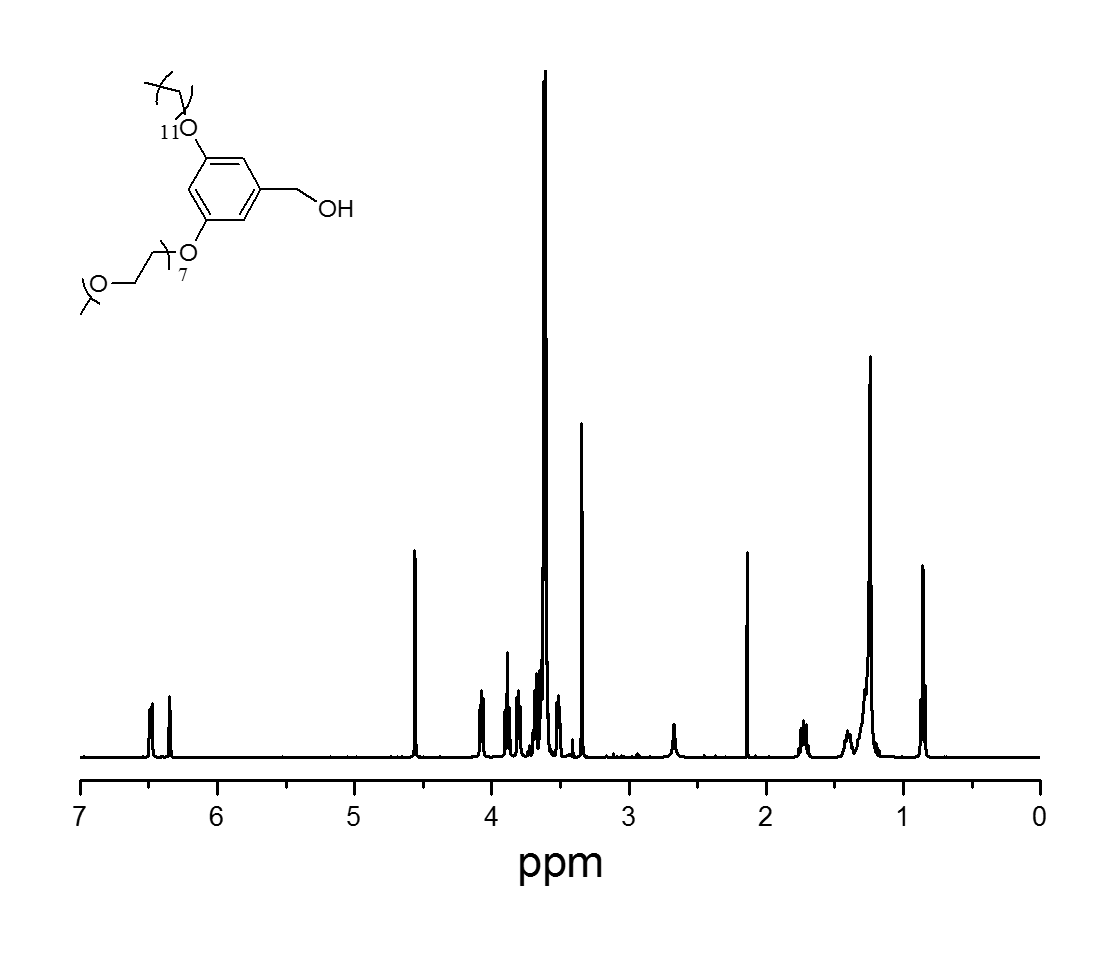

Supplement: Supplementary file 3 — Additional file 3: Figure S3. Component (5) 3-(dodecyloxy)-5-(EO7)phenol. 1H NMR (400 MHz, CDCl3, δ): 0.86–0.9 (t, 3H, –CH3); 1.26–1.35 (m, 16H, –CH2–); 1.39–1.46 (m, 2H, –CH2–); 1.72–1.79 (m, 2H, –CH2–); 3.37 (s, 3H, –CH3); 3.53–3.55 (m, 2H, –CH2–); 3.63–3.68 (m, 28H, –CH2–); 3.69–3.72 (m, 2H, –CH2–); 3.82–3.85 (t, 2H, –CH2–); 3.9–3.93 (t, 2H, –CH2–); 4.1–4.12 (t, 2H, –CH2–); 4.6 (s, 2H, –CH2–); 6.37–6.38 (t, 1H, –C6H3–); 6.51–6.53 (d, 2H, –C6H3–). The 1H NMR spectrum of component (5) 3-(dodecyloxy)-5-(EO7)phenol. [file 12951_2018_423_MOESM3_ESM.tif]

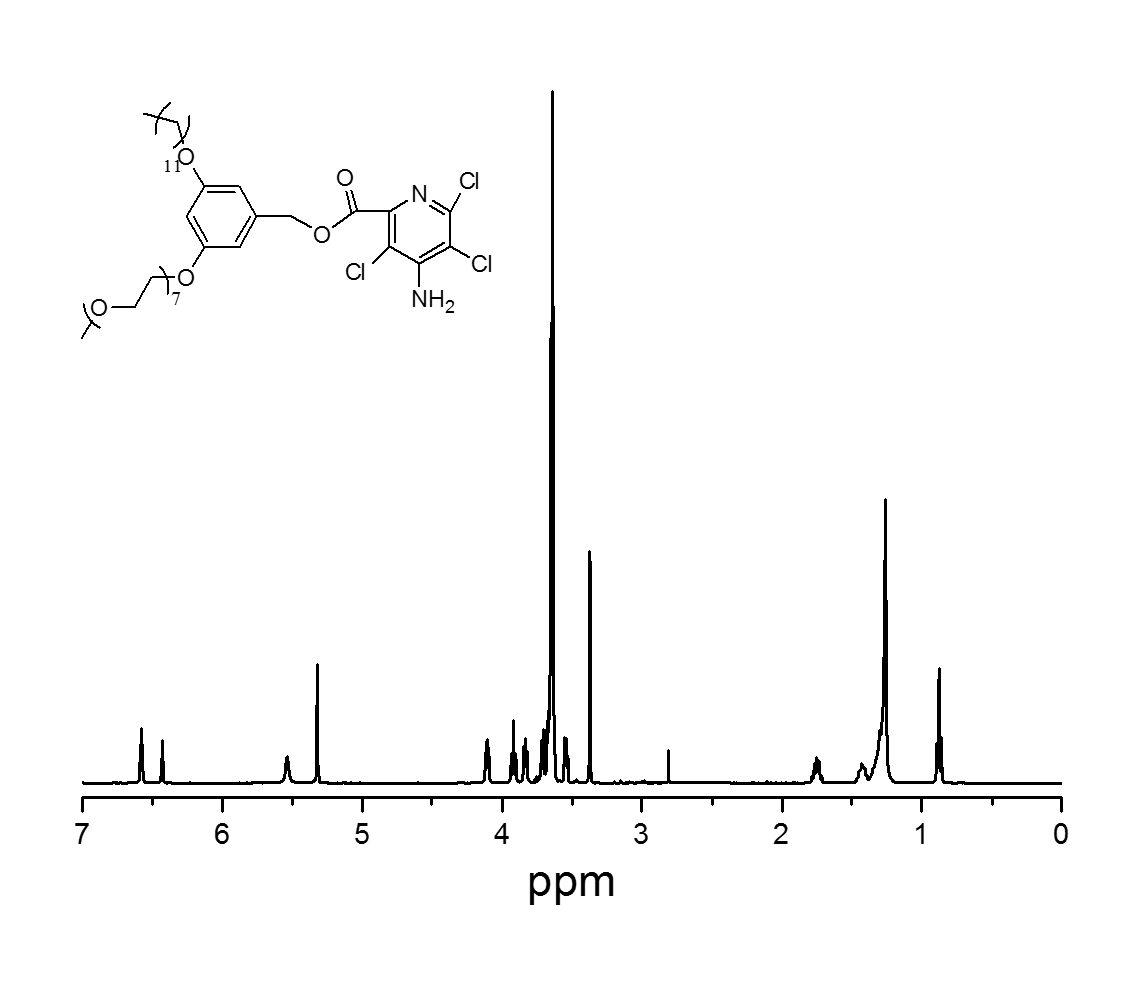

Supplement: Supplementary file 4 — Additional file 4: Figure S4. Component (6) 3-(dodecyloxy)-5-(EO7)benzyl-PIC. 1H NMR (400 MHz, CDCl3, δ): 0.85–0.89 (t, 3H, –CH3); 1.25–1.34 (m, 16H, –CH2–); 1.38–1.45 (m, 2H, –CH2–); 1.71–1.78 (m, 2H, –CH2–); 3.36 (s, 3H, –CH3); 3.52–3.55 (m, 2H, –CH2–); 3.63–3.68 (m, 4H, –CH2–); 3.71–3.73 (m, 2H, –CH2–); 3.82–3.84 (t, 2H, –CH2–); 3.89–3.92 (t, 2H, –CH2–); 4.09–4.11 (t, 2H, –CH2–); 5.31 (s, 2H, –CH2–); 5.53 (s, 2H, –NH2); 6.41–6.42 (t, 1H, –C6H3–); 6.56–6.57 (d, 2H, –C6H3–). The 1H NMR spectrum of component (6) 3-(dodecyloxy)-5-(EO7)benzyl-PIC. [file 12951_2018_423_MOESM4_ESM.tif]

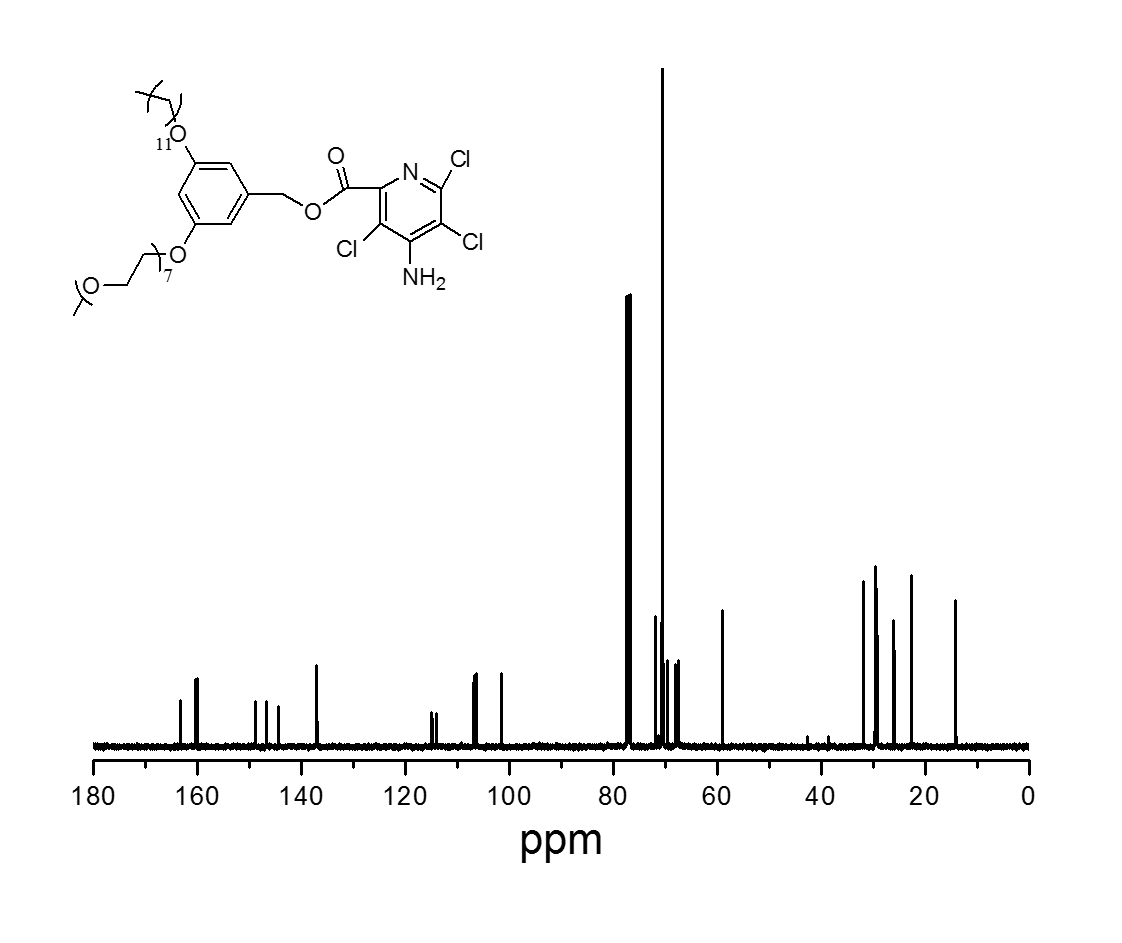

Supplement: Supplementary file 5 — Additional file 5: Figure S5. Component (6) 3-(dodecyloxy)-5-(EO7)benzyl-PIC. 13C NMR (400 MHz, CDCl3, δ): 14.13 (s); 22.68 (s); 26.03 (s); 29.21 (s); 29.34–29.39 (d); 29.58–29.66 (m); 31.91 (s); 59.02 (s); 67.49–68.12 (t); 69.63 (s); 70.49–70.79 (t); 71.91 (s); 76.79–77.42 (t); 101.57 (s); 106.32–106.83 (d); 114.06–114.94 (d); 137.06 (s); 144.39 (s); 146.74 (s); 148.96 (s); 160.04–160.41 (d); 163.33 (s). [file 12951_2018_423_MOESM5_ESM.tif]

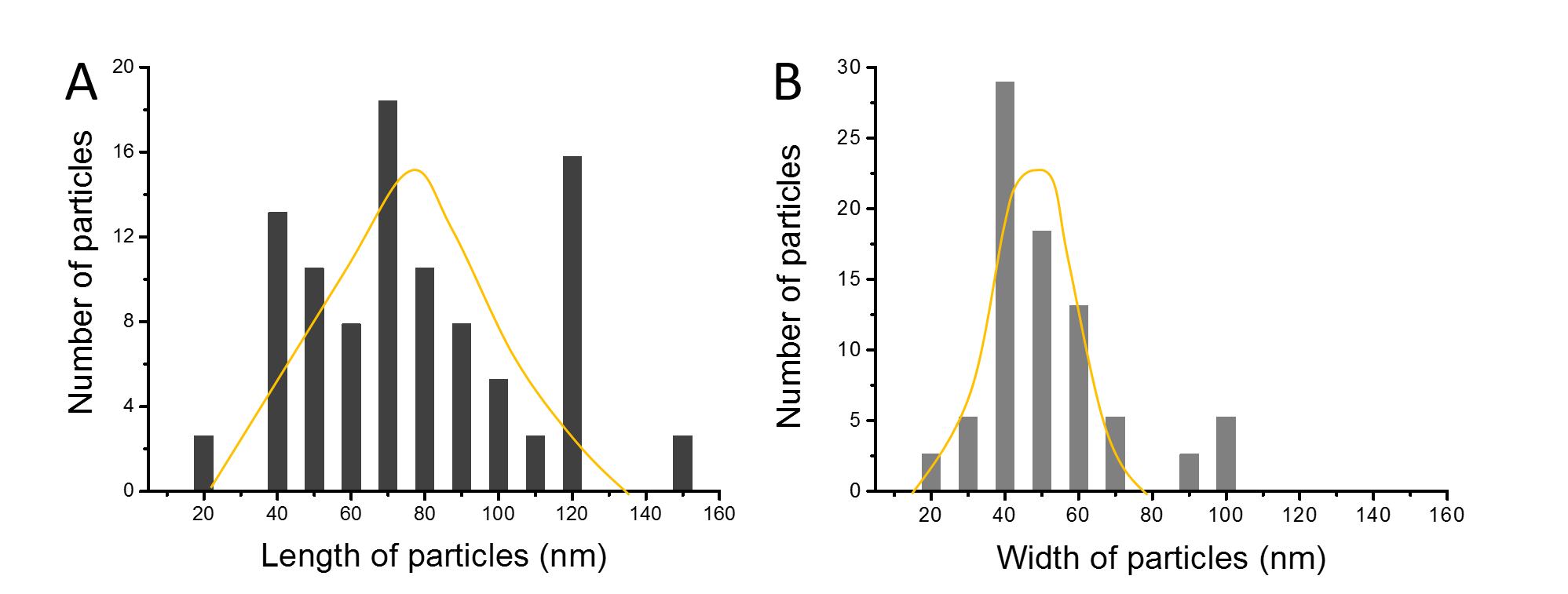

Supplement: Supplementary file 6 — Additional file 6: Figure S6. Size distribution of the SAP molecules. From the TEM images the length and width of SAP particle was estimated to be 82 ± 30 nm and 55 ± 17 nm respectively. Though TEM images are an indicative of smaller SAP conjugates, a through sampling regime can confirm the larger size of the aggregates. [file 12951_2018_423_MOESM6_ESM.tif]

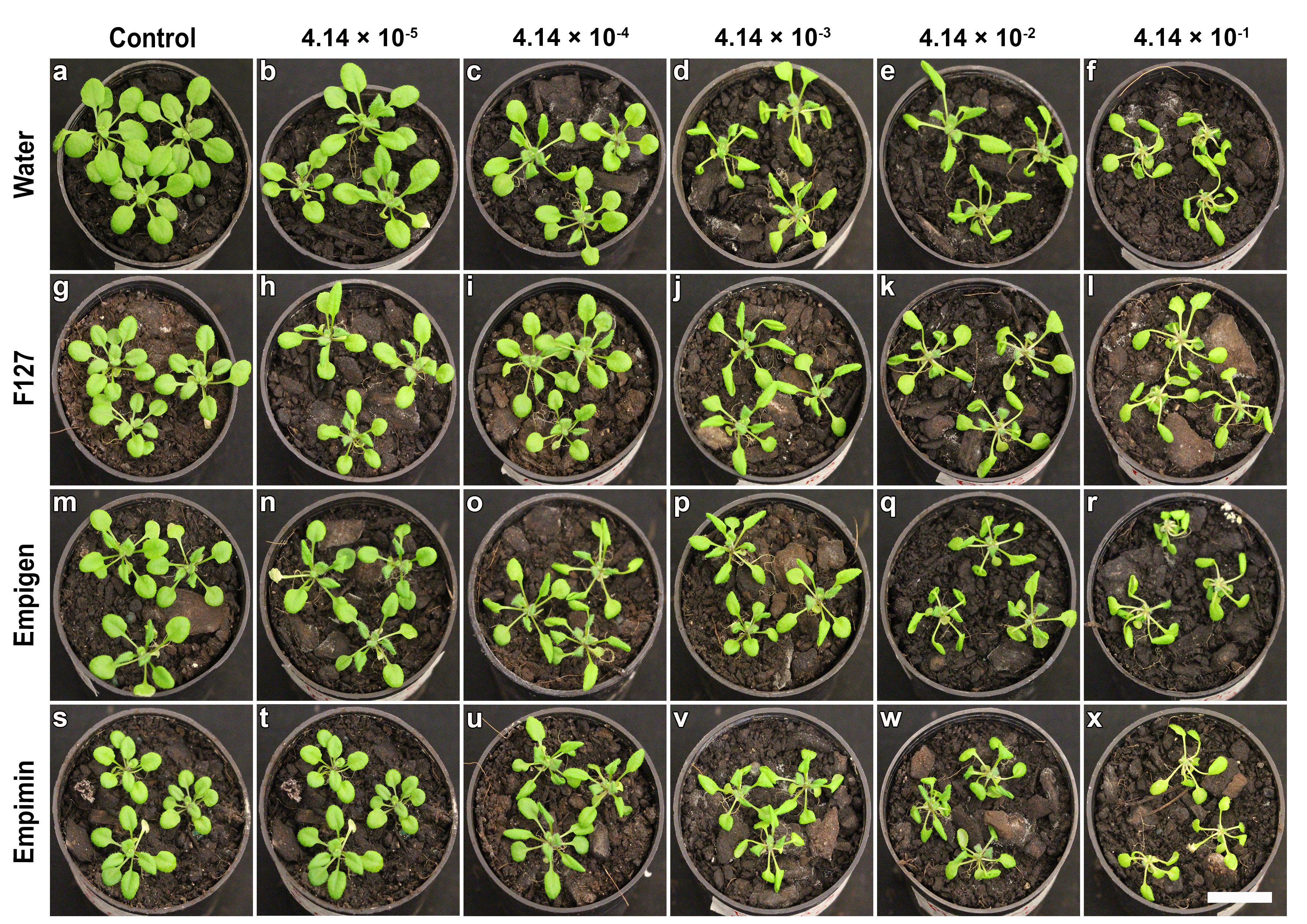

Supplement: Supplementary file 7 — Additional file 7: Figure S7. Surfactant-assisted delivery of picloram to A. thaliana. The image on the left hand side is labelled with relevant surfactant whose concentration was kept constant at 0.01% (w/v) while the respective concentrations of picloram were given on the top. (a) Control A. thaliana plant three days after water treatment. (b to f) The herbicidal effect intensified with the increase in the concentration of picloram (mixed in water) spray application and are marked by the presence of leaf curling (c to f), chlorosis (d to f) and petiole curling (f). (g to l) F127 based picloram treatments were similar to picloram (in water) treatments. (m to r) A stronger phytotoxicity effect on images (m to r) was evident for the treatments containing incremental doses of picloram in empigen. (s to x) For picloram delivered with empimin the herbicidal effect was similar to the same effect earlier observed for F127 based picloram delivery. Scale bar approximately equals to 1.5 cm. [file 12951_2018_423_MOESM7_ESM.tif]

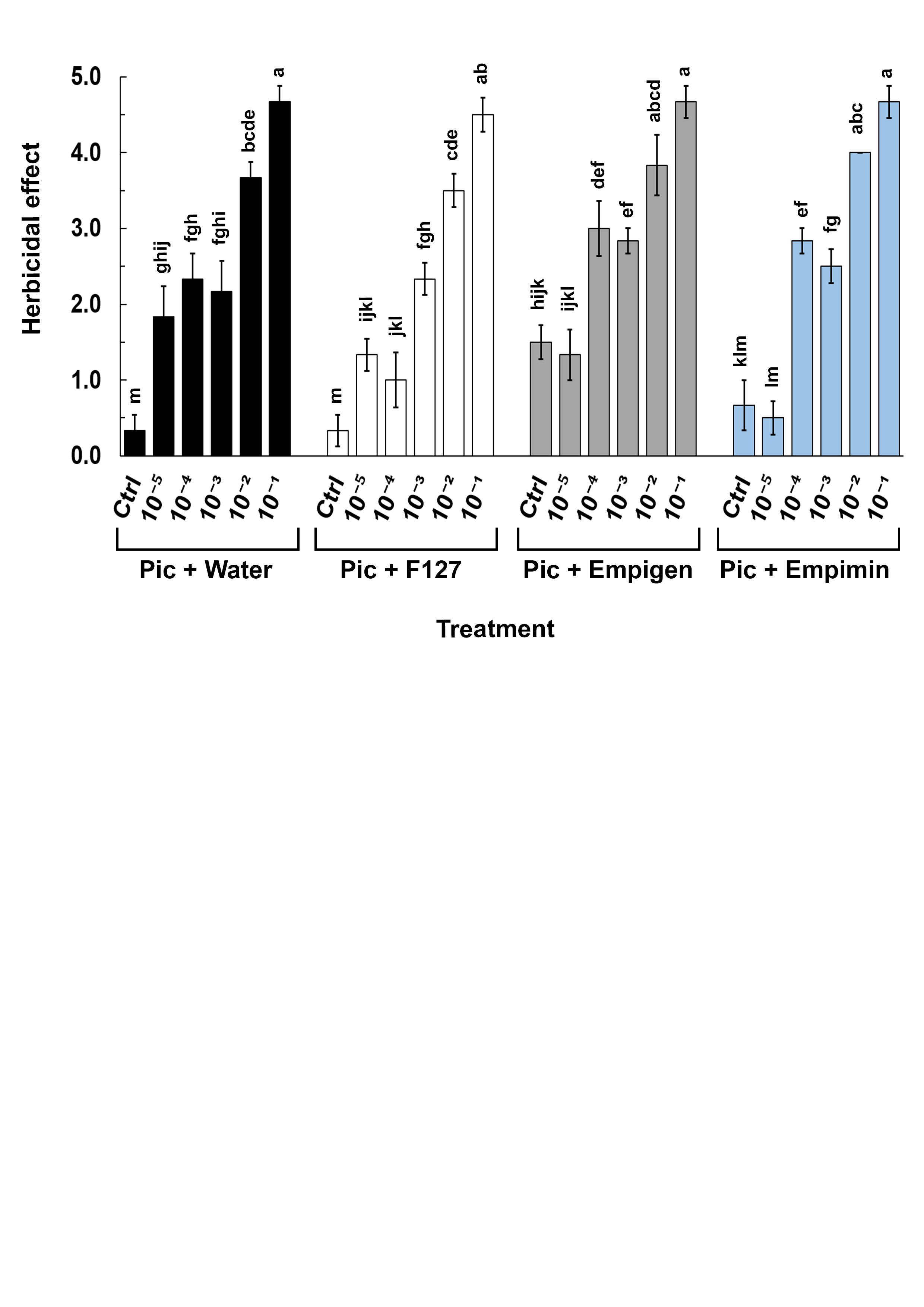

Supplement: Supplementary file 8 — Additional file 8: Figure S8. Herbicidal effect on A. thaliana after surfactant assisted picloram delivery. Three days after the application of picloram separately with water, F127, empimin and empigen, maximum herbicidal effect was observed for the highest concentration of picloram (10−1 mM) applied. Note that in the figure all x-axis concentrations are multiplied by 4.14, Ctrl is the control for the relevant picloram solvents. Ctrl = control, Pic = picloram. The bars ± SE labelled with the same letters are statistically similar (p < 0.05), Duncan’s posthoc test. [file 12951_2018_423_MOESM8_ESM.tif]

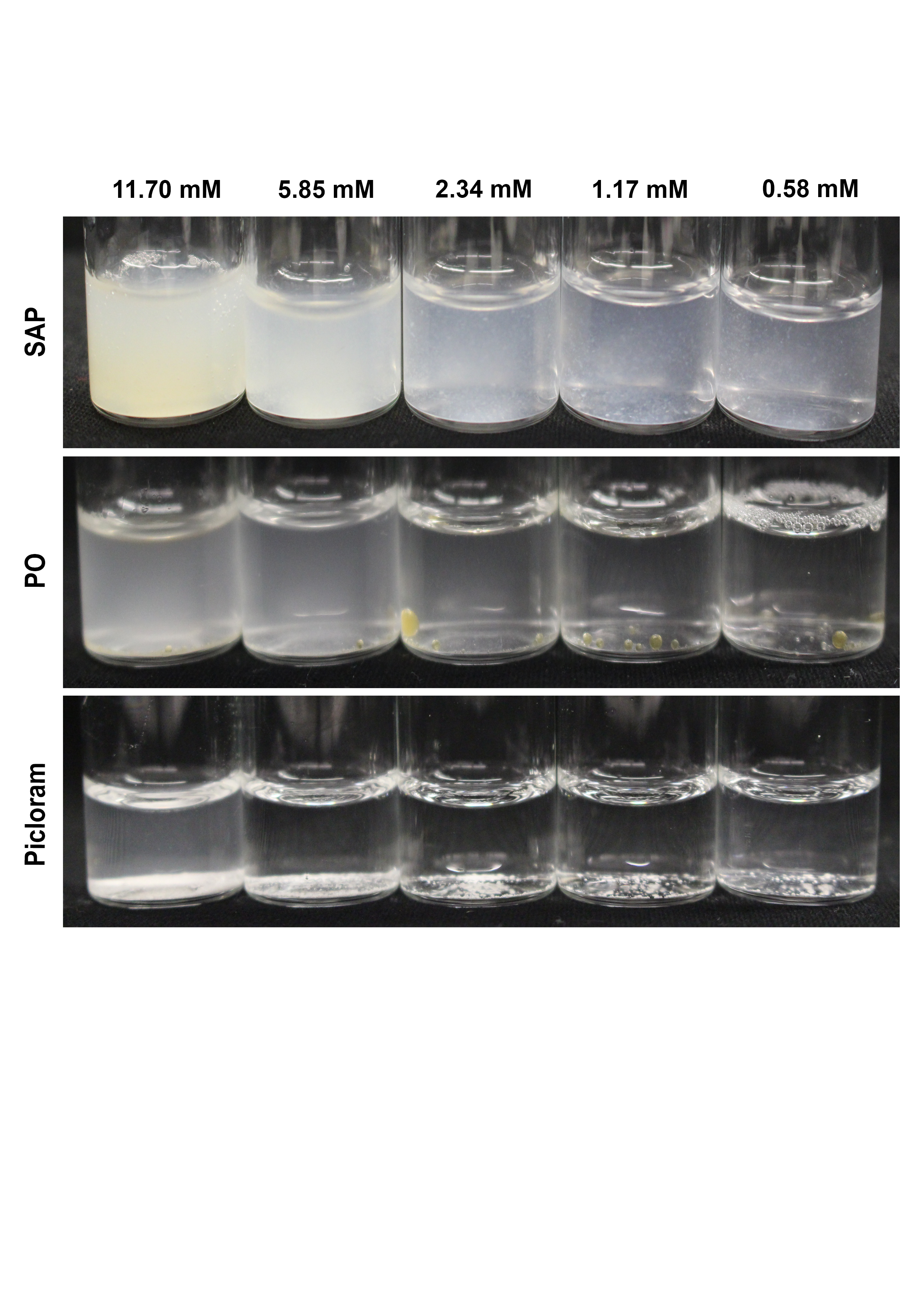

Supplement: Supplementary file 9 — Additional file 9: Figure S9. Solubility of SAP, PO and picloram in water. Self-assembly picloram when mixed in water formed a cloudy emulsion while PO and picloram at similar concentrations formed a precipitate. [file 12951_2018_423_MOESM9_ESM.tif]

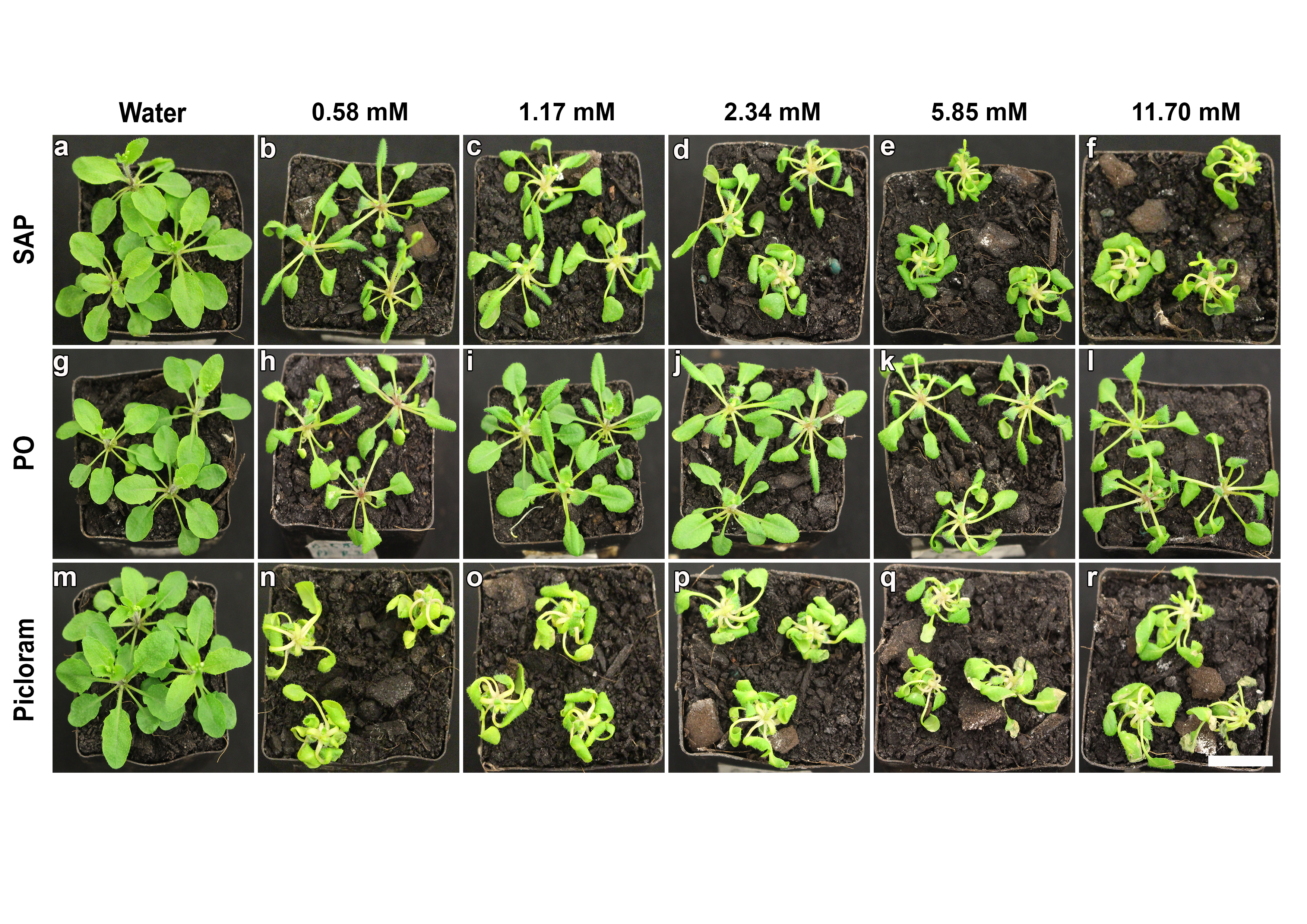

Supplement: Supplementary file 10 — Additional file 10: Figure S10. Effects of SAP delivery to plants. The images are labelled with relevant formulations on the left hand side with which they were spray applied while on the top are their respective concentrations. (a) Water treated healthy control plant three days after the spray treatments. The herbicidal effect of the SAP on plants was evident by the presence of leaf curling that elevated to petiole curling with the increase in dosage of SAP from (b) 0.58 mM through (c) 1.17 mM to (d) 2.34 mM. In addition to leaf and petiole curling, further increase in the concentration of SAP from 2.34 mM to (e) 5.85 mM and (f) 11.70 mM caused the leaves to twist along the main axis of the plant. (g) Water treated control for PO treatments. For PO applications at low concentrations, the phytotoxicity was limited to leaf curling (h to l) irrespective of the concentration. (m) A. thaliana plant treated with water. On the contrary, picloram applications induced a strong phytotoxicity marked by (n to p) the presence of chlorosis, petiole and leaf curling, (q and r) tissue necrosis and death. Scale bar equal to one cm. [file 12951_2018_423_MOESM10_ESM.tif]
